# Genome‐wide locus–allele comparison reveals differential evolution dynamics from annual wild to landrace and released cultivar soybeans

**DOI:** 10.1002/tpg2.70037

**Published:** 2025-05-14

**Authors:** Xinyang Hu, Jianbo He, Junyi Gai

**Affiliations:** ^1^ Soybean Research Institute, Zhongshan Biological Breeding Laboratory (ZSBBL), MARA National Center for Soybean Improvement & MARA Key Laboratory of Biology and Genetic Improvement of Soybean, State Innovation Platform for Integrated Production and Education in Soybean Bio‐Breeding, National Key Laboratory for Crop Genetics and Germplasm Enhancement, Jiangsu Collaborative Innovation Center for Modern Crop Production Nanjing Agricultural University Nanjing China

## Abstract

Previous studies on population evolution relied primarily on allele frequency analysis using molecular markers or genome sequence segments, like selective sweeps. With the sequencing technique developed, we suggest the genome‐wide locus–allele comparison to detect the genomic structure variation among populations. Its key point lies in taking SNP linkage disequilibrium block as uniform genomic marker for genome‐wide gene and inter‐gene regions to meet the requirement of multiple alleles in natural populations. A sample composed of 750 annual wild accessions (WAs), landraces (LRs), and released cultivars (RCs) of soybean from southern, northern, and northeastern China eco‐regions (SC, NC, and NEC, respectively) were analyzed for their evolution dynamics involving four evolutionary processes (WA→LR→RC, WA_SC_→WA_NC_→WA_NEC_, LR_SC_→LR_NC_→LR_NEC_, and LR_SC_→RC_SC_/LR_NC_→RC_NC_/LR_NEC_→RC_NEC_). Our major finding was the discovery of allele and locus zero/one variation between/among ancestor‐filial populations involving a large part of the whole population alleles and loci, 25.10% and 18.62% in domestication and modern breeding stages, respectively, which was not detected by selective sweeps. The essence of population evolution is the allele zero/one changes based on ordinary allele frequency changes, which causes the locus zero/one changes. The allele/locus zero/one variation happened more often when their frequency was at 0.0–0.3 and 0.8–0.99 in the previous stage generation, respectively. The WA and LR geographic evolution are different processes due to different combination of allele/locus zero/one changes by natural versus artificial selection pressures. Compared to per‐year allele exclusion, the rate of per‐year allele emergence is relatively stable in domestication and modern breeding (2.75E‐5 vs. 1.34E‐5 and 1.42E‐3 vs. 1.10E‐5), respectively.

## Core Ideas

- GLAC explores allele/locus zero/one changes among populations as evolution essence, neglected by selective sweeps.
- During domestication (WA→LR) and modern breeding (LR→RC) in CSGP, 79.45% and 71.94% wild alleles were inherited.
- In WA→LR, 13.74%/6.71% alleles and 15.36%/1.53% loci excluded/emerged, totally 25.10% alleles changed.
- In LR→RC, 14.22%/0.11% alleles and 15.56%/0.02% loci excluded/emerged, totally 18.62% alleles changed.
- WA and LR geographic evolution differ in allele/locus zero/one changes under natural versus artificial selection.

**Abbreviations**
GLAC: genome‐wide locus–allele comparison
IA: inherited allele
LD: linkage disequilibrium
LR: landrace
NC: northern China
NEC: northeastern China
RC: released cultivar
SC: southern China
SNP: single nucleotide polymorphism
SNPLDB: SNP linkage disequilibrium block
SSs: selective sweeps
WA: wild accession
XP‐CLR: cross‐population composite likelihood ratio

## 1 INTRODUCTION

**FIGURE 1:**
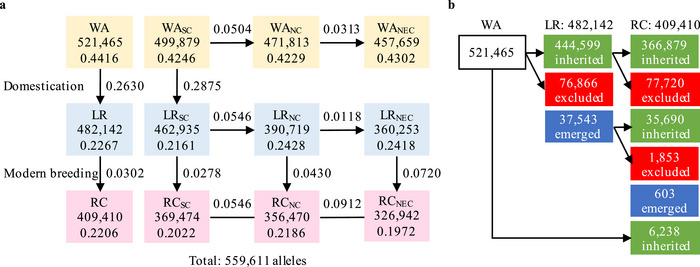
The genetic parameters of the population/subpopulation and allele changes in Chinese soybean evolutionary process. (a) The population/subpopulation allele number, genetic diversity (*π*) and genetic differentiation index (*F*_ST_) in Chinese soybean evolutionary process. The population/subpopulation allele number and genetic diversity (*π*) are in the box behind the population/subpopulation name, while the genetic differentiation statistics (*F*_ST_) are marked between boxes. (b) The allele inheritance and zero/one changes (exclusion and emergence) from WA to LR and then to RC. The green, red, and blue boxes mean inherited, excluded, and emerged alleles, respectively. LR, landrace; NC, northern China; NEC, northeastern China; RC, released cultivar; SC, southern China; WA, wild accession; WA_SC_, wild accession in southern China, and the similar for others.

The cultivated soybean (*Glycine max* [L.] Merr.) was domesticated from the annual wild soybean (*Glycine soja* Sieb. & Zucc.) in China about 5,000 years ago (Dashiell, 2005). During the long history, generations of farmers have continuously developed their landraces (LRs) for various types of soybean production and passed them to their offspring. Based on the historical farmers' LRs, the modern breeding for cultivars started about 100 years ago with a great number of modern cultivars released. Accordingly, three stages/types of the soybean germplasm with consanguinity, including wild accessions (WAs), farmers' LRs, and modern released cultivars (RCs), were collected and reserved in germplasm storages in China, serving as the ideal genetic materials for studying the evolutionary mechanisms in WA→LR→RC. The previous studies deduced that annual wild soybean originated from perennial wild soybean at low latitudes of the east part of the world, possibly in southern China (SC, north of 23.5° N) and then disseminated to northern latitudes in east Asia, including northern China (NC) and northeastern China (NEC) (Liu et al., 2021). The SC has been recognized as the center of origin for cultivated soybeans based on phylogenetic and clustering analyses using restriction fragment length polymorphism, random amplified polymorphic DNA, simple sequence repeat, and nucleotide diversity (Gai et al., 2000; J. Guo et al., 2010; Wen et al., 2009), where domestication happened by local farmers. Then its cultivation extended northward to adapt to northern conditions. Therefore, the geographic evolution/adaption of WA and LR was from SC to NC and then to NEC (SC→NC→NEC). After that, different eco‐regions set breeding programs for their modern cultivars adapted to local geographic‐climatic conditions. The key to elucidating the evolutionary mechanism lies in gaining insight into the genetic structure changes during WA→LR→RC processes, in geographic diversifications in SC→NC→NEC of WA and LR, and in different eco‐region local breeding progress. The evolutionary process outlines are shown in Figure 1a. Fortunately, the three‐stage populations of soybean germplasm with consanguinity in China (the center of origin) were collected and are available for a detailed study on their evolutionary mechanism.

Evolution is fundamentally the evolution of a population, in which a number of alleles or even genes are excluded or emerged, leading to qualitative changes in the genetic constitution of the population. In contrast, ordinary allele frequency changes primarily reflect quantitative shifts in existing alleles. Within a population's genome, an allele serves as an entity that determines phenotypic performance, while a gene or locus represents a position on a chromosome that may host multiple alleles performing different phenotypes. Therefore, characterizing population evolution is to characterize the variation of gene–allele constitution of the population, involving allele emergence or exclusion, rather than the ordinary allele frequencies.

However, before the high‐throughput genome‐wide resequencing developed in crops, the earlier studies could measure the ordinary allele frequency changes based on some microsatellites and/or a small number of nucleotide polymorphism markers. Accordingly, some genetic statistics, including genetic diversity (*π*) (Nei & Li, 1979), genetic differentiation index (*F*_ST_) (Weir & Cockerham, 1984), selection sweeps or cross‐population composite likelihood ratio (XP‐CLR) for selective signals (Chen et al., 2010; Zhou et al., 2015), relative identical by descent for introgressions (X. Wang et al., 2019), and genomic evolutionary rate profiling for deleterious mutations (Kim et al., 2021), were used in charactering the evolutionary processes.

The term “selective sweep” (SS) was introduced by Berry et al. (1991) to describe the phenomenon of diversity reduction caused by “genetic hitchhiking” in *Drosophila* population and has been generally used in detecting selection activities (Berry et al., 1991; Stephan, 2019). If the frequency of an allele increases in the population, the nearby linked alleles also increase in frequency. Such genetic hitchhiking leads to an SS that alters genetic diversity in the genomic region (Sabeti et al., 2006). The first genome‐wide detection of SSs was performed in humans (Akey et al., 2002) and *Drosophila melanogaster* (Harr et al., 2002; Schlotterer, 2002) to characterize the footprint of positive selection. The earliest genome‐wide detection of SSs in crops was in soybeans in 2010 to identify genomic regions related to domestication (Lam et al., 2010). Subsequently, SSs were widely studied to reveal the origin and to mine domestication genes in crops, such as rice (Huang et al., 2012; Molina et al., 2011; Xu et al., 2011), soybean (Kim et al., 2021; Lam et al., 2010; Zhou et al., 2015), cucumber (Qi et al., 2013), cotton (Fang et al., 2017; M. Wang et al., 2017), watermelon (S. Guo et al., 2019), lettuce (Wei et al., 2021), Chinese jujube (M. Guo et al., 2021), Tartary buckwheat (Zhang et al., 2021), and sorghum (Wu et al., 2022). SS detection is based on methods such as *π*, *F*_ST_, and XP‐CLR. The top 5% of genomic regions with the highest statistical values are treated as SSs. However, these methods did not account for variations in gene–allele composition and the presence of multiple alleles.

With the advancement of genome‐wide sequencing, the genome‐wide locus–allele comparison (GLAC) was used to study the evolutionary mechanism among populations. It was found that the information obtained from XP‐CLR or SS was mainly on ordinary allele frequency changes with the major information on allele and even locus exclusion and emergence ignored (Akbari et al., 2018; Field et al., 2016). In fact, to population evolution, the allele and locus exclusion and emergence (or zero/one changes) are variations in quality while the ordinary frequency change is only variation in quantity of a same allele. Thus, in the present study, the three‐stage populations with kinship were chosen for charactering the dynamics of the four evolutionary processes, that is, WA→LR→RC; WA_SC_→WA_NC_→WA_NEC_; LR_SC_→LR_NC_→LR_NEC_; and LR_SC_→RC_SC_, LR_NC_→RC_NC_, and LR_NEC_→RC_NEC_, especially their allele and locus exclusion and emergence (i.e., allele/locus zero/one variation) as qualitative changes of the population. For the four evolutionary processes, the dynamics for domestication and modern breeding, for natural selection in WA_subregion_ and artificial selection in LR_subregion_, and for LR_subregion_ to RC_subregion_ are all to be characterized. At the same time, the ordinary allele frequency variation in quantity changed into allele exclusion/existence in quality is also to be characterized. Here, we proposed the GLAC method based on genome‐wide sequencing of a whole population rather than part of them. The GLAC is in fact to trace the dynamic state of each allele of a locus, mainly on allele/locus zero/one changes in addition to ordinary allele frequency changes among populations/subpopulations. At first, it groups the tightly linked single nucleotide polymorphisms (SNPs) to generate uniform genomic markers with multi‐allelic haplotypes, designated SNPLDB (SNP linkage disequilibrium block; He et al., 2017), and then establishes and compares the locus–allele matrix to identify the allele (even locus) zero/one changes and ordinary allele frequency changes between/among populations with kinships. Thus, GLAC could gain insights into the soybean evolution mechanism. This is a tremendous statistic work, and the main results are expressed as statistics of the dynamic state of alleles and loci between/among populations/subpopulations.

**Core Ideas**

- GLAC explores allele/locus zero/one changes among populations as evolution essence, neglected by selective sweeps.
- During domestication (WA→LR) and modern breeding (LR→RC) in CSGP, 79.45% and 71.94% wild alleles were inherited.
- In WA→LR, 13.74%/6.71% alleles and 15.36%/1.53% loci excluded/emerged, totally 25.10% alleles changed.
- In LR→RC, 14.22%/0.11% alleles and 15.56%/0.02% loci excluded/emerged, totally 18.62% alleles changed.
- WA and LR geographic evolution differ in allele/locus zero/one changes under natural versus artificial selection.

## 2 MATERIALS AND METHODS

### 2.1 Plant materials

A total of 750 accessions in Chinese soybean germplasm pool (CSGP), including 127 WA, 424 LR, and 199 RC, were sampled from ∼20,000 accessions in the germplasm storage at the National Center for Soybean Breeding, Nanjing, China, based on their agricultural traits. These accessions originated from NEC, NC, and SC, which represent the three major soybean cultivation areas in China (Table S1).

### 2.2 Genomic DNA preparation and sequencing

Genomic DNA was extracted from young leaves using the cetyltrimethylammonium bromide protocol (Murray & Thompson, 1980). Sequencing libraries (approximately 350 bp sequencing libraries) were constructed according to the manufacturer's instructions. Genome sequencing was performed on an Illumina HiSeq X Ten sequencer at Vazyme, and paired‐end reads 150‐bp in length, with approximately 5x depth, were generated.

### 2.3 SNP calling and SNPLDB construction

After filtering the adaptor reads and low‐quality reads, clean sequence data were mapped to the reference genome *Wm82.a2.v1* (Schmutz et al., 2010) using BWA software (Version 0.6.1‐r104) (H. Li & Durbin, 2009) with default parameters. SNP calling was performed using GATK software (Version 2.4‐7‐g5e89f01) (McKenna et al., 2010) and SAMtools software (Version 0.1.18) (H. Li et al., 2009) with default parameters. Common SNPs identified by both methods with a minor allele frequency >1% were retained for further analysis. The SNPLDB markers with multiple haplotypes were constructed by using *rtm‐gwas‐snpldb* function in RTM‐GWAS software (Version 2020.0) with default parameters at linkage disequilibrium (LD) *D*′ ≥ 0.70, as a uniform genomic marker for gene and inter‐gene regions to meet the multi‐allele requirement in natural population (He et al., 2017). It was reported as the best separation point for genome sequences to include the whole‐genome genes and genetic elements (Gabriel et al., 2002).

### 2.4 Genetic differentiation and SS detection

The number of alleles in each subpopulation was counted. The VCFtools software (Version 0.1.15) (Danecek et al., 2011) was used to compute nucleotide diversity (*π*) and genetic differentiation index (*F*_ST_) between subpopulations at each SNPLDB locus. The overall average of all loci was considered as the whole‐genome parameter. The XP‐CLR method was used to detect SSs between subpopulations (Chen et al., 2010) (https://reich.hms.harvard.edu/software).

Evidence for selection across the genome during domestication and modern breeding was evaluated using two comparisons: LR versus WA for domestication and RC versus LR for modern breeding. A sliding window of 100 kb with a 10 kb step was used for genome‐wide SNP markers. Adjacent windows with high statistics were grouped into single regions to represent the effects of a single SS. The top 5% of genomic regions with the highest statistical values were considered SSs.

### 2.5 GLAC

By comparing the SNPLDB allele matrices between the ancestral population and its derived populations, the excluded, emerged, and inherited alleles (IAs) were identified. The excluded and emerged alleles were coded allele zero/one changes, and the IAs were further classified into three cases: IA_i_ with frequency of 1 (locus fixation), IA_ii_ with frequency increased to 1 or first decreased from 1 (locus polymorphism disappeared or emerged), and IA_iii_ with ordinary frequency increased or decreased.

## 3 RESULTS

### 3.1 Allele inheritance, exclusion, and emergence in WA→LR→RC, with allele exclusion/emergence designated the allele zero/one variation

In this study, we re‐sequenced 750 soybean accessions (in CSGP) with approximately 5x depth, including 127 WA, 424 LR, and 199 RC originated from SC, NC, and NEC, as a representative sample composed of the three stages (WA, LR, and RC) of soybean germplasm populations, involving the four evolutionary processes (Figure 1a; Table S1).

Resequencing of these accessions by an Illumina HiSeq X Ten sequencer generated a total of 22 billion 150‐bp paired‐end reads (3.3 Tb of sequences), with an average coverage of 94.16%. After mapping against the soybean Williams 82 reference genome (Schmutz et al., 2010), there were 2,745,637 SNPs with a minor allele frequency >1% (Table S2). From this, a total of 154,088 genome‐wide SNPLDBs (He et al., 2017) with 559,611 alleles (Table S3) were constructed at LD *D*′ ≥ 0.70, as a uniform genomic marker for gene and inter‐gene regions to meet the multi‐allele requirement in natural population. It was reported as the best separation point for genome sequences to include the whole‐genome genes and genetic elements (Gabriel et al., 2002). The allele numbers of these SNPLDBs were between 2 and 27 with an average of 3.6 alleles on each locus (Table S3); however, 90.61% loci with allele numbers between 2 and 7, 6.66% loci with allele numbers between 8 and 10, and only 2.73% loci with allele numbers >10 (Table S4).

To observe the divergence among the 750 accessions, we performed a principal component analysis for whole‐genome SNPLDBs (Figure S1a–c). The most of the WAs clustered together, with a few close to some LRs. In cultivated soybeans, LRs and RCs formed a subclade within a larger mixed clade. A phylogenetic tree through whole‐genome SNPLDBs provided similar results (Figure S1d), with cultivated soybeans forming a tight cluster that is clearly separate from wild soybeans. These results suggested that there were larger genetic changes during domestication. Here, many LRs are closed to RCs, and some WAs are located in LR clades. This indicates that among the WA, LR, and RC groups, there are variations within each group, even with overlaps between WA and LR and between LR and RC, especially the latter because of the related pedigree, short breeding history, and inter‐infiltration among eco‐regions. In addition, there appeared a few WAs with vining growth, black seeds but larger and leaf areas collected from wild field.

From the SNPLDB data set of all the 750 accessions and the component subsets for different eco‐regions, the inherited, excluded, and emerged alleles during WA→LR→RC were counted and shown in Figure 1b. However, the allele exclusion and allele emergence, the locus exclusion and locus emergence (locus polymorphism disappeared and emerged), designated allele zero/one changes, and locus zero/one changes along with ordinary allele frequency changes are shown in Table 1. These various allele/locus changes involving three different stages of germplasm populations (WA, LR, and RC) made the cases complicated due to cross‐section happened.

**TABLE 1:** The allele changes during WA→LR→RC compared to those from selective sweeps.

Evolutionary process	Allele		Allele in selective sweeps	
Allele type	Allele number	%	Allele number	% in each type
***LR vs. WA (domestication)***				
Inherited	444,599	79.45		
Frequency of 1	37	0.01	10	27.03
Frequency changes	444,562	79.44		
Increase to 1 (LPD)	**23,670**	**4.23**	**5127**	**21.66**
Decrease from 1 (LPE)	**2351**	**0.42**	**357**	**15.19**
Ordinary frequency increase	206,020	36.81	19,174	9.31
Ordinary frequency decrease	212,521	37.98	22,339	10.51
Excluded	**76,866**	**13.74**	**13,227**	**17.21**
Emerged	**37,543**	**6.71**	**2969**	**7.91**
Frequency of 0	603	0.11	68	11.28
***RC vs. LR (modern breeding)***				
Inherited	402,569	71.94		
Inherited (WA from LR)	366,879	65.56		
Inherited (LR emerged)	35,690	6.38		
Frequency of 1	22,261	3.98	2,286	10.27
Frequency changes	380,308	67.96		
Increase to 1 (LPD)	**23,971**	**4.28**	**3488**	**14.55**
First decrease from 1 (LPE)	**37**	**0.01**	**5**	**13.51**
Restored decrease from 1 (LPR)	1409	0.25	77	5.46
Ordinary frequency increase	175,203	31.31	11,843	6.76
Ordinary frequency decrease	179,688	32.11	14,110	7.85
Excluded	**79,573**	**14.22**	**9245**	**11.62**
Emerged	**603**	**0.11**	**52**	**8.62**
Inherited from WA as direct parent in RC	6238	1.11	293	4.70
Frequency of 0	70,628	12.62	6,890	9.76

*Note*: Allele exclusion and emergence are coded as allele zero/one changes, and these may further cause locus zero/one changes or locus polymorphism disappeared (LPD) and locus polymorphism emerged (LPE). Notably, only an allele frequency first decrease from 1 was coded as LPE. If these loci had polymorphism in more initial population/subpopulation, their frequency restored decrease from 1 was coded as locus polymorphism restored (LPR). “%” means the corresponding alleles accounting for the total allele number (559,611) of the whole population, while “% in each type” means the corresponding alleles accounting for that of the allele type. LR versus WA means the comparison of LR to WA and the same is true for others. Frequency of 1 or 0 means that these alleles frequency is keeping in 1 or 0, without zero/one and frequency change. Boldface signifies the important outcomes.

Abbreviations: LR, landrace; RC, released cultivar; WA, wild accession.

Here, during domestication and modern breeding, the allele number decreased from 521,465 in WA (93.18% of 559,611) to 482,142 in LR (86.16%) and then to 409,410 in RC (73.16%) (Figure 1a). In domestication, 444,599 (79.45%) alleles were inherited from WA to LR, and 76,866 (13.74%) and 37,543 (6.71%) alleles were excluded and emerged, respectively (Table 1). However, in modern breeding, 366,879 (65.56%) alleles from WA through LR to RC, plus 35,690 (6.38%) LR‐emerged alleles to RC, in a total of 402,569 (71.94%) alleles inherited from LR to RC, and 6,238 (1.11%) alleles directly inherited from WA to RC (as direct parents), and 79,573 (14.22%) and 603 (0.11%) alleles excluded and emerged in RC, respectively. Thus, between the two stages of domestication (WA→LR) and modern breeding (LR→RC), the allele exclusion amounts were about similar, but the allele emergence numbers were quite different (37,543 vs. 603). If the per‐year rate is concerned, 15.37 versus 795.73 alleles/year were excluded for domestication and modern breeding, respectively (Table 2), indicating quite more selection pressure in modern breeding. As for the per‐year allele emergence rate, 7.51 versus 6.03 alleles/year for domestication and modern breeding, respectively, indicated that the mutation rate was about the same for the two evolution stages (1.34E‐5 vs. 1.10E‐5).

**TABLE 2:** The allele/locus zero/one changes during domestication and modern breeding.

Variation type	Domestication	Per‐year	Modern breeding	Per‐year
Allele exclusion	76,866 (13.74%)	15.37 (2.75E‐5)	79,573 (14.22%)	795.73 (1.42E‐3)
Allele emergence	37,543 (6.71%)	7.51 (1.34E‐5)	603 (0.11%)	6.03 (1.10E‐5)
Locus polymorphism disappeared	23,670 (15.36%)	4.73 (3.07E‐5)	23,971 (15.56%)	239.71 (1.56E‐3)
Locus polymorphism emerged	2351 (1.53%)	0.47 (3.06E‐6)	37 (0.02%)	0.37 (2.00E‐6)

*Note*: The percentage of allele zero/one changes was relative to a total of 559,611 alleles, and that of locus zero/one changes was relative to a total of 154,088 loci. The data were summarized from Table 1. In calculating per‐year rate, 5000 years and 100 years are used for domestication and modern breeding, respectively.

### 3.2 Allele changes may cause locus polymorphism disappeared and emerged, designated as locus zero/one variation

The excluded and emerged alleles are considered as allele zero/one changes, while an allele excluded on a two‐allele locus (or n‐1 allele on an n‐allele locus excluded), the polymorphism or this locus was disappeared. On the other hand, if an allele (or more) emerged on a genome segment originally without polymorphism, then a new locus formed. Therefore, with the allele zero/one variation happened, a part of them might cause automatically their locus polymorphism disappeared (locus excluded) or cause a segment first with polymorphism (locus emerged), which is designated locus zero/one variation. Accordingly, an IA might meet the following cases: IA with frequency of 1 (no polymorphism or kept fixation, coded IA_i_), IA with frequency increased to 1 or first decreased from 1 (polymorphism disappeared or emerged or zero/one variation, coded IA_ii_), and IA with ordinary frequency increase or decrease (ordinary allele frequency change, IA_iii_) in the evolution processes. Notably, only the frequency first time decreased from 1 was coded as locus polymorphism emerged. If the locus had polymorphism in an initial population, but the present population with segment frequency decreased from 1, it was coded as locus polymorphism restored.

For IA_i_, during WA→LR→RC, there were 37 (0.01%) and 22,261 (3.98%) alleles with frequency of 1 (kept without polymorphism) in domestication and modern breeding, respectively (Table 1). For IA_ii_, there were 23,670 (4.23%) and 23,971 (4.28%) alleles with frequency increased to 1 (polymorphism disappeared) in domestication and modern breeding, respectively. However, there were 2,351 (0.42%) and 37 (0.01%) alleles in which frequency first decreased from 1 (increased polymorphic loci). These allele changes caused 15.36% (23,670 in 154,088) and 15.56% (23,971) loci disappeared, and 1.53% (2,351) and 0.02% (37) loci newly emerged in the two stages, respectively (Table 2). For IA_iii_, there were 206,020 (36.81% of 559,611) and 175,203 (31.31%) alleles with ordinary frequency increase, whereas 212,521 (37.98%) and 179,688 (32.11%) alleles with ordinary frequency decrease in the two stages, respectively (Table 1). From WA→LR and then to LR→RC, the disappeared locus number was similar, but the per‐year number was quite different (4.73 vs. 239.71 loci/year), indicating the disappeared loci number inflated largely during modern breeding (Table 2). However, the emerged locus number was quite different (603 vs. 37), but the per‐year emerged locus number was similar in domestication and modern breeding (0.47 vs. 0.37 loci/year), indicating the locus mutation rate was similar for the two evolution stages (3.06E‐6 vs. 2.00E‐6). The locus emergence per‐year rate was lower than the allele emergence per‐year rate in a number rank (3.06E‐6 and 2.00E‐6 vs. 1.34E‐5 and 1.10E‐5).

From the above, locus zero/one variations are important genetic constitution changes in the evolution processes. During WA→LR→RC, the disappeared locus number was always more than the emerged locus number, which might cause the fixed locus number continuously increasing from 2388 (1.55%) to 23,707 (15.39%) and then to 46,232 (30.00%) (Table 2).

### 3.3 Allele successive changes from domestication to modern breeding lead to both permanent allele exclusion and presence

Considering that domestication and modern breeding are two continuous evolutionary processes, the successive allele changes during WA→LR→RC were inspected (Table S5). The 91.88% (70,628 in 76,866) of domestication excluded alleles did not appear in RC or permanently excluded. However, 95.06% (35,690 in 37,543) of domestication mutant emerged alleles kept polymorphism and further inherited to RC in modern breeding. Furthermore, 79,573 modern breeding excluded alleles (Table 1) were traced to 94.81% (75,446 in 79,573) of alleles with frequency decreased already during domestication (Table S5), and 23,971 alleles with frequency increased to 1 (or allele fixation) in modern breeding (Table 1) were traced to 98.33% (23,571 in 23,971) of alleles with frequency increased already during domestication (Table S5). These observations suggest that the allele selection direction in domestication and modern breeding was probably the same to cause allele fixation.

In addition, a large number of loci lost their polymorphism in WA→LR and may further lose polymorphism in LR→RC, for example, 22,261 loci (14.45%) permanently lost their polymorphism in domestication and are kept without polymorphism in modern breeding (Table S6). However, there are 23,571 (15.30%) loci with allele frequency already increased during domestication and then further increased to 1 in modern breeding (locus polymorphism disappeared). These two genetic phenomena accounted for 99.13% (22,261 plus 23,571 in 46,232) of the fixed loci in RC, indicating that the allele fixation caused further locus fixation.

### 3.4 Genome‐wide position and often happened frequency in previous stage generation of allele/locus zero/one changes in WA→LR→RC

**FIGURE 2:**
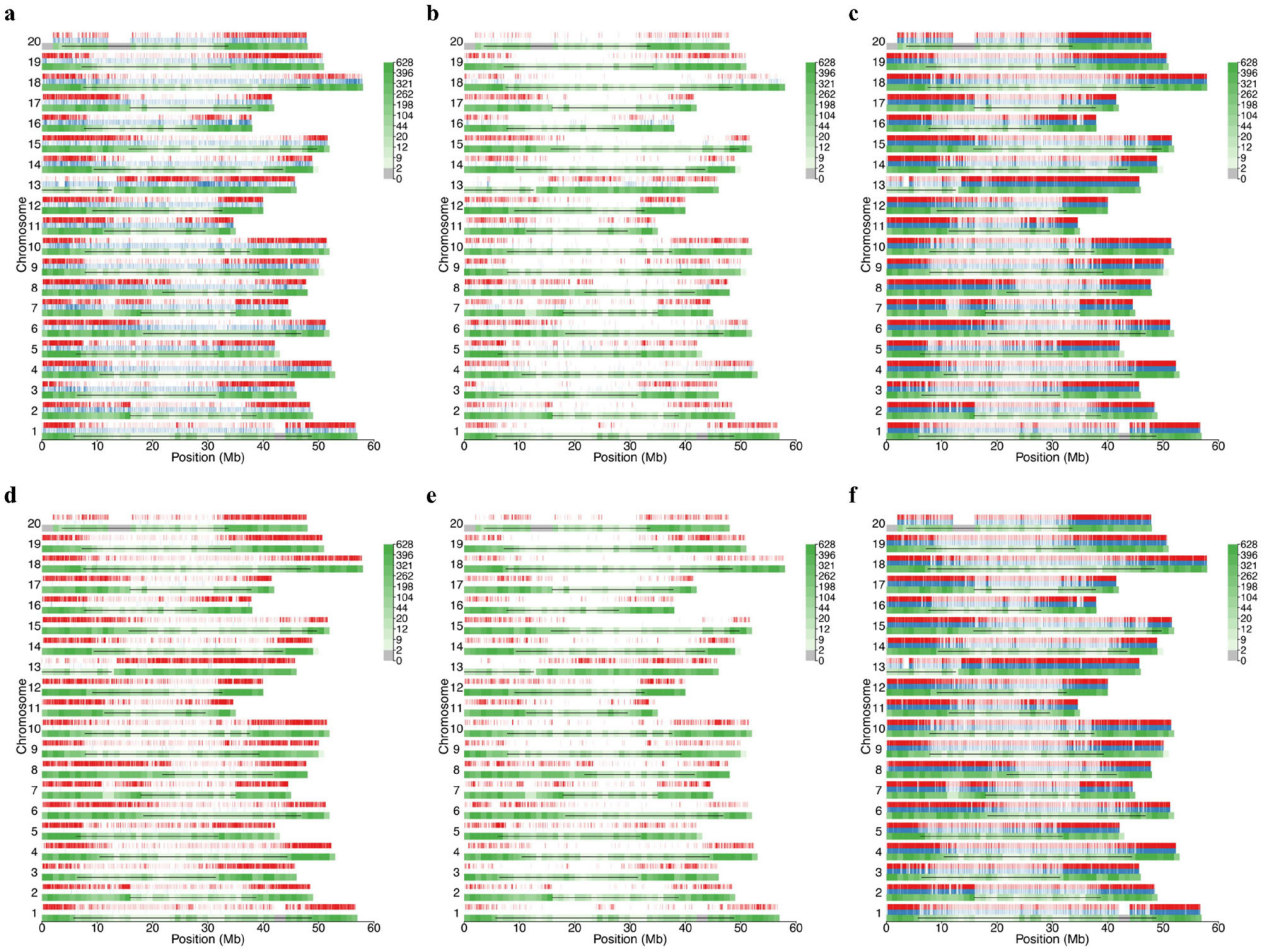
The genome‐wide position of zero/one and ordinary frequency changes during domestication and modern breeding. (a and d) The whole genome position of allele zero/one changes during domestication and modern breeding, respectively. Here, the red and blue bars show the position of the excluded and emerged alleles, respectively. In addition, the green bar shows locus density at the genome position, and the black horizontal solid line indicates pericentromeric region, and the same for others. Most of the excluded and emerged alleles (red and blue bars) were located in chromosome arm regions, while the emerged allele number was more than the excluded allele number in pericentromeric regions, which was different from that in the whole genome. Here, the position of modern breeding emerged alleles is not expressed because of the small number. (b) and (e) The whole genome position of locus zero/one changes during domestication and modern breeding, respectively. Here, the red and blue bars show the position of polymorphism disappeared and emerged locus, respectively. Most of the locus zero/one changes (red and blue bars) were located in chromosome arm regions. Similarly, the position of modern breeding emerged loci is also not expressed because of the small number. (c) and (f) The whole genome position of allele ordinary frequency changes during domestication and modern breeding, respectively. Here, the red and blue bars show the position of ordinary frequency increase and decrease alleles, respectively. Most of the allele ordinary frequency changes (red and blue bars) were located in chromosome arm regions.

The large number of allele/locus zero/one during domestication and modern breeding are distributed on chromosomes differentially. In Figure 2, the red and blue bars show the position of alleles or loci excluded and emerged, respectively; the green bar shows the locus density as the locus background with the black horizontal solid line indicating pericentromeric regions. The allele zero/one variations were located in chromosome arm regions (Figure 2a,d), with the emerged allele number more than the excluded allele number in pericentromeric regions. Here, the position of modern breeding emerged alleles is not marked because of its small number. Similarly, the locus zero/one variations changed during domestication and modern breeding, like allele zero/one changes (red and blue bars) were located also mainly in chromosome arm regions (Figure 2b,e). Here the position of modern breeding emerged loci is also not marked because of its small number. As for the ordinary frequency changes (Figure 2c,f), the red and blue bars show the position of ordinary frequency increase and decrease alleles, respectively. The most of ordinary allele frequency changes (red and blue bars) were located in chromosome arm regions. Obviously, the ordinary allele frequency changes were many more than the others (Figure 2c,f compared to Figure 2a,d and b,e), which is the basis for allele/locus zero/one changes. The above results indicate that the allele frequency changes, therefore, the allele/locus zero/one changes are mainly located in genome‐wide arm regions. This might be due to the pericentromeric regions having less genes.

The often‐happened allele frequency at previous stage population was observed for allele/locus zero/one variation in the present stage population. Table 3 shows the frequency distribution of allele/locus zero/one changes in WA→LR. (i) In “0” row, the 76,866 excluded alleles (20,998 + 37,678 + 13,559 + …. + 7) in LR were from alleles in WA with frequency 0.0–0.8 with most part (20,998 + 37,678 + 13,559) from 0.0 to 0.3 (this row coded as “top edge” of the table). (ii) In “0” column, the 37,543 emerged alleles (34,735 + 2,383 + 322 + …. + 3) in LR were of allele frequency 0.0–0.7 with most part (34,735 + 2,383 + 322) at 0.0–0.3 in LR (this column coded as “left edge” of the table). (iii) In “1” row, the 23,670 excluded loci (5,912 + 11,460 + 4,451 + …. + 1) in LR had their frequency 0.1–0.99 in WA with most part (5,912 + 11,460 + 4,451) at 0.7–0.99 (this row coded as “bottom edge” of the table). (iv) In “1” column, the 2,351 emerged loci (2,243 + 104 + 4) had their frequency 0.7–0.99 with most part (2,243 + 104) at 0.8–0.99 in LR (this column coded as “right edge” of the table). Moreover, there was a large amount of allele frequency increase to 0.9–0.99 and decrease to 0.0–0.1 in LR during domestication (these alleles were not symmetrical between the two sides of the diagonal).

**TABLE 3:** The frequency distribution of alleles from wild accession (WA) to landrace (LR).

Allele number		Class limit of allele frequency in WA											
		0	0.0–0.1	0.1–0.2	0.2–0.3	0.3–0.4	0.4–0.5	0.5–0.6	0.6–0.7	0.7–0.8	0.8–0.9	0.9–0.99	1
Class limit of allele frequency in LR	**0**	603	**20,998**	**37,678**	**13,559**	**3303**	**1008**	**265**	**48**	**7**			
	**0.0–0.1**	**34,735**	84,372	57,296	34,668	16,139	9253	4400	1777	802	226	10	
	**0.1–0.2**	**2383**	20,182	7516	4734	2734	2087	1344	760	431	152	6	
	**0.2–0.3**	**322**	10,739	5225	3188	2120	1669	1189	697	418	154	20	
	**0.3–0.4**	**90**	5858	3895	2,340	1621	1367	1074	678	409	181	42	
	**0.4–0.5**	**9**	3,896	3,291	1837	1,305	1111	956	604	438	193	52	
	**0.5–0.6**	**1**	1815	2755	1760	1177	1216	1017	796	643	312	90	
	**0.6–0.7**	**3**	1191	2709	1861	1422	1375	1240	875	725	451	156	
	**0.7–0.8**		595	2,596	1,864	1,366	1482	1277	1075	933	675	353	**4**
	**0.8–0.9**		127	2098	2124	1446	1510	1434	1389	1459	1387	1288	**104**
	**0.9–0.99**		14	1218	3070	2894	3730	5264	6714	10,923	15,319	13,875	**2243**
	**1**			**1**	**8**	**21**	**120**	**505**	**1192**	**4451**	**11,460**	**5912**	37

*Note*: “0” row: “top edge” of the table, excluded alleles (frequency most from 0.0 to 0.3 in WA); “0” column: “left edge” of the table, emerged alleles (frequency most from 0.0 to 0.3 in LR); “1” row: “bottom edge” of the table, excluded loci (frequency most from 0.7 to 0.99 in WA); “1” column: “right edge” of the table, emerged loci (frequency most from 0.8 to 0.99 in LR). Moreover, there were a large amount of alleles frequency increase of 0.9–0.99 and decrease of 0.0–0.1 in LR during domestication. Boldface signifies the important outcomes.

Table 4 shows the frequency distribution of allele/locus zero/one changes in LR→RC. The often‐happened allele frequency in previous stage population was narrower than those in WA→LR. The 79,573 excluded alleles (top edge) in RC were mainly with their frequency 0.0–0.1 in LR; the 603 emerged alleles (left edge) in RC mainly had their frequency 0.0–0.1 in RC. The 23,971 excluded loci (bottom edge) in RC were mainly with their frequency 0.9–0.99 in LR; the 37 emerged loci (right edge) in RC were mainly with their frequency 0.9–0.99 in RC.

**TABLE 4:** The frequency distribution of alleles from landrace (LR) to released cultivar (RC).

Allele number		Class limit of allele frequency in LR											
		0	0.0–0.1	0.1–0.2	0.2–0.3	0.3–0.4	0.4–0.5	0.5–0.6	0.6–0.7	0.7–0.8	0.8–0.9	0.9–0.99	1
Class limit of allele frequency in RC	**0**	70,628	**79,484**	**89**									
	**0.0–0.1**	**588 + 6184**	142,746	16,122	2433	248	15						
	**0.1–0.2**	**15 + 54**	17,442	15,396	7502	2080	393	32					
	**0.2–0.3**		3453	7799	8157	4679	1937	427	61	1			
	**0.3–0.4**		507	2343	5181	5020	3846	1330	376	22			
	**0.4–0.5**		45	486	1915	3621	3482	2421	1219	351	52		
	**0.5–0.6**		1	93	457	1517	2503	2904	2690	1268	288	9	
	**0.6–0.7**			1	69	361	1204	2824	3665	2696	1121	197	
	**0.7–0.8**				27	29	293	1378	2717	3859	2993	1045	
	**0.8–0.9**						19	260	1132	3115	5179	4163	**1 + 14**
	**0.9–0.99**							6	148	908	4729	35,883	36 + 1,395
	**1**										**4**	**23,967**	22,261

*Note*: “0” row: “top edge” of the table, excluded alleles (frequency most from 0.0 to 0.1 in LR); “0” column: “left edge” of the table, emerged and directly inherited (from WA) alleles (frequency most from 0.0 to 0.1 in RC); “1” row: “bottom edge” of the table, excluded loci (frequency most from 0.9 to 0.99 in LR); “1” column: “right edge” of the table, emerged and restored loci (frequency most from 0.9 to 0.99 in RC). Boldface signifies the important outcomes.

Abbreviation: WA, wild accession.

From the above, the allele exclusion in LR happened mainly at their frequency 0.0–0.3 in WA, while in RC, it happened at their frequency 0.0–0.1 in LR, indicating that more selection pressure happened in domestication. The allele emergence happened with their frequency 0.1–0.3 in LR, while it happened at their frequency 0.0–0.1 in RC. The locus exclusion in LR happened at their allele frequency 0.7–0.99 in WA, while it happened in RC at their allele frequency 0.9–0.99 in LR, indicating more selection pressure in domestication. The locus emergence happened in LR with their allele frequency 0.8–0.99 in LR, while it happened in RC at the partner allele frequency down from 1 to 0.9–0.99 in RC. Thus, the excluded and emerged loci were basically those with two alleles, when an allele with its frequency of 1, this locus was disappeared, while a segment with a mutation happened (its partner changed from frequency 1), this caused a new locus emerged.

### 3.5 Evolutionary importance of allele/locus zero/one changes relative to ordinary frequency changes and SSs

Table 5 shows that in domestication (WA→LR) the allele/locus zero/one changes (excluded allele, emerged allele, excluded locus, and emerged locus) accounted for 13.74% + 6.71% + 4.23% + 0.42% = 25.10% (relative to 559,611 alleles in the whole population), whereas the ordinary frequency changes (increase plus decrease) accounted for 36.81% + 37.98% = 74.79%. And in modern breeding (LR→RC), the allele/locus zero/one changes accounted for 14.22% + 0.11% + 4.28% + 0.01% = 18.62%, whereas the ordinary frequency accounted for 31.31% + 32.11% = 63.42% of the total population allele number. Since the allele/locus zero/one changes involve the presence/non‐presence of allele/locus, they are genetic structure changes or qualitative changes, but the ordinary allele frequency change does not involve presence/non‐presence changes or only quantitative changes of a same allele. From the above text (Tables 3 and 4), the ordinary allele frequency changes varied from 0.0 to 0.99, and only alleles with a part of frequency (0.0–0.3 or 0.7–0.99) may cause allele/locus zero/one changes. Therefore, the importance of allele/locus zero/one changes is absolutely dominant over the ordinary allele frequency changes in evolutionary processes.

**TABLE 5:** The evolutionary dynamics of populations and subpopulations in terms of allele/locus zero/one and ordinary frequency changes (%).

Evolutionary Process	Restoration (allele + allele*)	Emergence (allele + allele*)	Exclusion (allele + allele*)	Ordinary freq. change (increase + decrease)	No change (zero + one)
LR vs. WA	–	6.71 + 0.42	13.74 + 4.23	36.81 + 37.98	0.11 + 0.01
RC vs. LR	1.11 + 0.25 (WA)	0.11 + 0.01	14.22 + 4.28	31.31 + 32.11	12.62 + 3.98
WA_NC_ vs. WA_SC_	–	2.38 + 0.25	7.40 + 0.96	39.92 + 40.23	8.29 + 0.57
WA_NEC_ vs. WA_NC_	3.27 + 0.56 (WA_SC_)	1.47 + 0.14	7.27 + 1.01	37.52 + 36.98	10.95 + 0.82
LR_NC_ vs. LR_SC_	1.97 + 0.52 (WA)	0.28 + 0.02	15.15 + 4.17	30.01 + 28.33	15.03 + 4.53
LR_NEC_ vs. LR_NC_	4.56 + 1.25 (WA, LR_SC_)	0.14 + 0.01	10.14 + 2.45	25.83 + 22.70	25.48 + 7.45
RC_SC_ vs. LR_SC_	1.25 + 0.23 (WA, LR)	0.07 + 0.00	18.03 + 5.36	27.48 + 26.78	15.95 + 4.83
RC_NC_ vs. LR_NC_	3.86 + 0.87 (WA, LR)	0.08 + 0.01	10.06 + 2.62	21.80 + 22.36	26.24 + 7.83
RC_NEC_ vs. LR_NEC_	5.34 + 1.15 (WA, LR)	0.09 + 0.01	11.39 + 3.20	19.34 + 20.55	30.19 + 8.74

*Note*: Each evolutionary motivator was expressed as the percentage of the corresponding alleles accounting for the total alleles (559,611). Restoration (allele + allele*): alleles directly inherited from other source(s) (such as WA used as direct parents), plus inherited alleles that caused locus polymorphism restored. Emergence (allele + allele*): emerged mutant alleles, plus inherited alleles with their frequency first decreased from 1 (loci emerged). Exclusion (allele + allele*): excluded alleles, plus inherited alleles with their frequency increased to 1 (loci excluded). Ordinary freq. change (increase + decrease): ordinary frequency increase and decrease changes. No change (zero + one): the alleles keeping frequency of 0 or 1 without zero/one and frequency changes.

Abbreviations: LR, landrace; NC, northern China; NEC, northeastern China; RC, released cultivar; SC, southern China; WA, wild accession; WA_SC_, wild accessions in southern China and the similar for others.

In addition, different loci might have experienced varied selection pressures, some with both allele zero/one (quality) and ordinary frequency (quantity) changes and some with only one of the two. During domestication and modern breeding, 28.78% and 24.21% loci had both allele zero/one and ordinary frequency changes, respectively (Table S7). However, 16.89% and 15.58% loci had only allele zero/one changes, while the other 54.31% and 44.85% loci had only ordinary frequency changes in the two stages, respectively, with the former being important although the latter being dominant. These observations suggest that there are complementary events between the qualitative zero/one and quantitative ordinary frequency changes.

To profile the evolutionary motivators from WA to LR and from LR to RC, the allele changes and locus changes have to be combined together (or expressed on a same scale of allele changes); we grouped excluded alleles and IAs with frequency increased to 1 (loci excluded) into exclusion (selection against, exclusion of allele + locus, or allele + allele*), and similarly grouped emerged alleles and IAs with frequency first decreased from 1 (loci emerged) into emergence (mutation, emergence of allele + locus, or allele + allele*) (Table 5). Moreover, directly IAs and IAs with frequency restored decreased from 1 (loci restored) were grouped into restoration (allele + locus or allele + allele*). In domestication, the major evolutionary motivator was exclusion (17.97% = 13.74% + 4.23%), emergence the next (7.13% = 6.71% + 0.42%), as qualitative changes of the genome, with the dominant part of ordinary frequency changes (increase + decrease, 74.79% = 36.81% + 37.98%) as quantitative changes of the genome (Table 5). However, the major evolutionary motivator in modern breeding was also exclusion (18.50% = 14.22% + 4.28%), emergence the next but very rare (0.12% = 0.11% + 0.01%), with the dominant part of ordinary frequency changes (63.42% = 31.31% + 32.11%) and those of restoration from WA (1.36% = 1.11% + 0.25%). It should be emphasized that 15.36% and 15.56% loci disappeared in domestication and modern breeding, respectively (Table 2), which were very important changes in evolution. In addition, another evolutionary motivator is genetic recombination to break down linkage obstacles based on the above genomic changes.

SSs (or signals of selection) in domestication and modern breeding of soybean may not explore the key evolutionary phenomena. To identify potential SSs during soybean domestication (WA vs. LR) and modern breeding (LR vs. RC), the genomic regions with extreme allele frequency spectra were scanned using XP‐CLR under 5% selection pressure (Chen et al., 2010). A total of 140 (79.15 Mb) domestication‐related and 150 (74.83 Mb) modern breeding–related genomic regions (Figure S2; Tables S8 and S9) were detected.

Comparing the location of the allele changes and SSs, it was found that only 17.21% of excluded alleles, 7.91% of emerged mutant alleles during domestication, 11.62% of excluded alleles, and 8.62% of emerged mutant alleles during modern breeding were located in SS regions (Table 1). Similarly, only a few locus zero/one changes are situated in the SS regions. These observations suggest that the allele/locus zero/one changes along with ordinary allele frequency changes happen on the whole genome rather than only on its small part (5%). Thus, the allele/locus zero/one changes or the qualitative changes of genetic structure could not be identified in SSs.

### 3.6 Dynamic genetic structure changes among geographic subpopulations in WA versus LR

Using the allele/locus zero/one analysis, the dynamic genetic structure changes among geographic subpopulations in WA were inspected, with the results listed in Table 5. There were 7.40% and 7.27% excluded alleles, 2.38% and 1.47% emerged alleles, 0.96% and 1.01% IAs with frequency increased to 1 (locus excluded), 0.25% and 0.14% IAs with frequency first decreased from 1 (locus emerged), 39.92% and 37.52% ordinary frequency increase alleles, and 40.23% and 36.98% ordinary frequency decrease alleles in WA_SC_→WA_NC_ and WA_NC_→WA_NEC_, respectively.

Compared to those in LR_SC_→LR_NC_ and LR_NC_→LR_NEC_ in Table 5, there were 15.15% and 10.14% excluded alleles, 0.28% and 0.14% emerged alleles, 4.17% and 2.45% IAs with frequency increased to 1 (locus excluded), 0.02% and 0.01% IAs with frequency first decreased from 1 (locus emerged), 1.97% and 4.56% directly IAs, 30.01% and 25.83% ordinary frequency increase alleles, and 28.33% and 22.70% ordinary frequency decrease alleles in LR_SC_→LR_NC_ and LR_NC_→LR_NEC_, respectively.

The geographic subpopulation evolution of WA was mainly a natural selection and adaptation process while the geographic subpopulation evolution of LR was an artificial selection accompanied with geographic selection and adaptation process; the former took hundreds of thousands of years while the latter took only several thousand years. However, there were many differences in allele/locus zero/one changes between the two evolutionary processes, many more emerged alleles/loci but less excluded alleles/loci, and more ordinary allele frequency increase and decrease in the WA natural evolution process (WA_SC_→WA_NC_→WA_NEC_), or in other words, less alleles/loci emergence but more alleles/loci exclusions, and less ordinary allele frequency increase and decrease in LR artificial modified with natural evolution process (LR_SC_→LR_NC_→LR_NEC_). Thus, the latter evolutionary process is characterized with less allele emergences and more allele exclusions due to artificial selection, including allele direct and indirect exclusion (which causes the locus emergence and exclusion).

In addition, in Tables S10 and S11, at both WA_SC_→WA_NC_ and WA_NC_→WA_NEC_ stages, the excluded alleles (top edge) were with their frequency 0.0–0.6 (focused on 0.0–0.2); the emerged alleles (left edge) had their frequency 0.0–0.6 (focused on 0.0–0.2). The excluded loci (bottom edge) were with their frequency 0.4–0.99 (focused on 0.9–0.99); the emerged loci (right edge) were mainly with their frequency 0.8–0.99 (focused on 0.9–0.99). In Tables S12 and S13, at both LR_SC_→LR_NC_ and LR_NC_→LR_NEC_ stages, the excluded alleles (top edge) were with their frequency 0.0–0.3 (focused on 0.0–0.1); the emerged alleles (left edge) had their frequency 0.0–0.4 (focused on 0.0–0.1). The excluded loci (bottom edge) were with their frequency 0.6–0.99 (focused on 0.9–0.99); the emerged loci (right edge) were mainly with their frequency 0.7–0.99 (focused on 0.9–0.99). Thus, the allele/locus zero/one change frequencies were wider in the WA subpopulation by natural evolution than in the LR subpopulation by artificial plus natural evolution.

### 3.7 Genetic structure changes of modern breeding in each eco‐region

The modern breeding in an eco‐region is mainly based on local LRs due to the differential requirement for adaptive maturity groups. There are three modern breeding major eco‐regions, including SC, NC, and NEC eco‐regions. The three modern breeding systems are characterized jointly with their allele/locus changes (Table 5). These include a small number of emerged alleles (0.07%, 0.08%, and 0.09%), a large number of excluded alleles (18.03%, 10.06%, and 11.39%), a small number of IAs caused by emerged loci (0.00%, 0.01%, and 0.01%), a large number of IAs caused by excluded loci (5.36%, 2.62%, and 3.20%), and a large number of ordinary allele frequency increase and decrease (27.48%, 21.80%, and 19.34% for increase and 26.78%, 22.36%, and 20.55% for decrease), respectively. It has been only about hundred years for modern breeding, therefore, only a very small number of mutation or allele emergence happened. But the allele exclusion happened many times in a short period, including locus zero/one variation (mainly caused by allele exclusion), which indicates the artificial selection pressure must be relatively strong in modern breeding processes.

From the above, among the three eco‐regions, modern breeding in NEC as well as NC was more enhanced than in SC, which was characterized relatively with more emerged allele/locus kept, less allele/locus excluded, and less ordinary allele frequency increased and decreased. As for the allele frequency distribution in LR_SC_ for allele/locus zero/one changes in RC_SC_ (Table S14), the excluded alleles (top edge) were with their frequency 0.0–0.3 (focused on 0.0–0.1); the emerged alleles (left edge) had their frequency 0.0–0.2 (focused on 0.0–0.1). The excluded loci (bottom edge) were with their frequency 0.7–0.99 (focused on 0.9–0.99); the emerged loci (right edge) were mainly with their frequency 0.8–0.99 (focused on 0.9–0.99), while those in LR_NC_→RC_NC_ and LR_NEC_→RC_NEC_ (Tables S15 and S16) were wider than those in LR_SC_→RC_SC_ (Table S14), indicating more breeding effort caused broader distribution in the former two eco‐regions than in the later eco‐region for allele/locus zero/one changes.

## 4 DISCUSSION

### 4.1 Characterization of the GLAC in exploring evolutionary mechanism compared to SSs

As indicated in Section 3, in addition to the ordinary allele frequency changes, the GLAC procedure can identify the allele zero/one changes, locus zero/one changes, and the fixation of alleles and loci among stage populations with ancestor–progeny relationship, which is the qualitative changes of the populations and neglected in SS analysis as well as other population genetic indices, such as genetic diversity, population differentiation, LD, and so forth (J. Guo et al., 2010; Hyten et al., 2007; Kim et al., 2021; Lam et al., 2010; Y. Li et al., 2008; Zhou et al., 2015).

In GLAC, the key points are the utilization of SNPLDB genomic markers and a reasonable sample of the materials that cover four evolutionary stage populations. The soybean genome is composed of about 50,000 genes, and 50,000 of inter‐genes accounted for about 20% and 80% of the genome sequences, respectively. To have a uniform marker for both gene and inter‐gene regions representing the whole genome and fitting the multiple allele requirements in a natural germplasm population, the SNPLDB marker is used to detect the genetic changes of the genome. A SNPLDB marker is a segment of genome sequence for which the genome is separated based on the LD threshold of *D*′ ≥ 0.7 (He et al., 2017), while haplotypes were treated as alleles of a SNPLDB. Fortunately, based on this kind of uniform genomic markers, the whole genome allele/locus zero/one changes can be exactly compared. Without this kind of uniform marker system, it is difficult to identify the allele/locus zero/one changes over the whole genome in populations with multiple alleles on each locus.

This study used a sample of soybeans covering the four evolutionary processes, that is, WA_SC_→WA_NC_→WA_NEC_, WA→LR→RC, LR_SC_→LR_NC_→LR_NEC_, and LR_SC_→RC_SC_/LR_NC_→RC_NC_/LR_NEC_→RC_NEC_. With this sample, all the allele/locus zero/one changes were identified, the allele/locus fixation was detected, the different evolution mechanisms between domestication (WA→LR) and the modern breeding (LR→RC) were explored, and the different geographic evolution mechanisms between WA_subregion_ and LR_subregion_ were distinguished.

For examining the reliability and persuasiveness of the GLAC method, the identified functional genes from a review paper by Hu et al. (2023) on seed weight/size are used to check our GLAC and SS results (Table S17). The change of seed weight/size from small seeded wilds to large seeded cultivars is an important domestication trait, and larger seed weight/size is also selected in cultivars by breeders. They summarized 31 genes identified and functionally tested for seed weight/size in previous studies. Among the 31 functional genes, two genes (2/31 = 6.45%) are located in our WA→LR SS regions, and six genes (6/31 = 19.35%) are located in our LR→RC SS regions, in a total of seven genes (with one duplicated for the two stages, 7/31 = 22.58%) located in our SS regions. Whereas in GLAC, 30 genes (96.77%) could be identified in our SNPLDB system of the GLAC method and all seven genes identified by SSs could be identified by our GLAC method. In fact, all the 31 genes can be found with GLAC method if the gene segment was used as markers under whole‐genome sequencing condition. These results indicate that our GLAC method may have more power and higher resolution to identify the domestication and evolution events.

### 4.2 Essence of population evolution and motivator differentiation in domestication versus modern breeding and natural selection versus artificial selection

The essence of population evolution in fact is the allele zero/one changes that cause the locus zero/one changes in populations in addition to the allele inheritance (allele passing down). Thus, the major motivators are allele/locus emergence and allele/locus exclusion. These are changes of genetic structure changes in quality, while the ordinary allele frequency changes are changes in quantity of a same allele, which might be the basis of allele/locus zero/one changes.

Some previous studies discussed the genetic dynamics of some traits in combination with the allele effect value (Fu, Wang, Ren, Du, Wang, et al., 2020; Fu, Wang, Ren, Du, Yang, et al., 2020; Liu et al., 2021). However, they only consider the allele exclusion and emergence (zero/one) changes with locus polymorphism zero/one changes concealed. Here in WA→LR→RC, the motivator pressure differentiated between domestication and modern breeding with the former having 76,866 alleles (13.74% or 15.37 alleles/year) and 23,670 loci (15.36% or 4.73 loci/year) excluded and 37,543 alleles (6.71% or 7.51 alleles/year) and 2,351 loci (1.53% or 0.47 loci/year) emerged (Table 2). However, in modern breeding, 79,573 alleles (14.22% or 795.73 alleles/year) and 23,971 loci (15.56% or 239.71 loci/year) were excluded and 603 alleles (0.11% or 6.03 alleles/year) and 37 loci (0.02% or 0.37 loci/year) emerged. That means in domestication, with the large number of alleles and loci excluded, also a large number of new alleles with some loci emerged. However, in modern breeding, the alleles and loci exclusion were accelerated, therefore, artificial breeding accelerated the genetic changes of the RC population.

Regarding the geographic diversifications from SC to NC and then to NEC in WA and LR, these two evolution processes were subjected to different selection pressures, that is, the natural and artificial plus geographic selection, respectively. As indicated in Section 3 (Table 5), less emerged alleles/loci and ordinary allele frequency changes but more excluded alleles/locus happened in LR artificial plus geographic diversifications than those in WA natural geographic diversifications). It indicates less years caused less allele/locus emergence, and artificial selection caused more allele/locus exclusion in LR geographic diversifications. Thus, the WA and LR geographic evolution are different processes due to different combinations of allele/locus zero/one changes as well as ordinary allele frequency changes caused by different selection pressure sources as well as their acting longevity.

In addition, regarding the three regional modern breeding processes, they are characterized jointly with a large number of allele and locus exclusion, and a small number of allele and locus emergence even in only about hundred years.

### 4.3 Relative stability of allele emergence compared to allele exclusion

As indicated in the above text, the basic evolution motivators are allele emergence and allele exclusion, from which locus exclusion and locus emergence happened, and all the qualitative changes are based on the quantitative changes of ordinary allele frequency. In plant breeding, breeders hope to obtain optimal genetic structure variations through new elite allele emergence and/or through undesirable allele exclusion as well as their resultant allele recombination. Table 2 shows that during domestication and modern breeding, the per‐year allele and locus exclusion rate varied between the two stages, but the per‐year allele and locus emergence rate kept at a same rank (1.34E‐5 vs. 1.10E‐5 for allele and 3.06E‐6 vs. 2.00E‐6 for locus). It means the per‐year allele and locus emergence rates are both relatively stable in domestication and modern breeding. It is basically in accordance with the natural mutation rate. This point implies that breeders could not expect more allele and locus emergence, especially elite allele emergence for their breeding progress. In other words, the breeders have to take measures to accelerate the new allele emergence, or as the recombination breeding, to utilize the allele recombination based on excluding inferior alleles for their breeding programs.

In summary, the present study developed the GLAC procedure to detect the genetic structure variation among populations with kinship for exploration of population evolution mechanism. The key point of the procedure is using SNPLDB as uniform genomic marker for gene and inter‐gene regions to meet the requirement of multiple alleles in natural populations. The sample consisted of 750 WA, LR, and RC accessions from SC, NC, and NEC eco‐regions involving four evolutionary processes, which were analyzed for their evolution dynamics. The major finding was the discovery of allele/locus zero/one variation in addition to the ordinary allele frequency changes, which were not possible in SSs and other genetic statistic indicators. The allele/locus zero/one changes among populations in the four evolutionary processes were identified, and based on the results, the evolutionary mechanism in different evolution processes was explored. In conclusion, the GLAC procedure can identify the evolution motivators through counting the allele and locus exclusion and emergence in exact quantity and can utilize the relative constitution of allele/locus zero/one variation to distinguish the differentiation of population evolution. However, there is another important motivator to be quantified, that is, recombination or LD among loci, which is to be further studied.

## AUTHOR CONTRIBUTIONS

**Xinyang Hu**: Conceptualization; data curation; formal analysis; investigation; writing—original draft; writing—review and editing. **Jianbo He**: Conceptualization; data curation; investigation; supervision; writing—review and editing. **Junyi Gai**: Conceptualization; funding acquisition; investigation; methodology; project administration; supervision; writing—review and editing.

## CONFLICT OF INTEREST STATEMENT

The authors declare no conflicts of interest.

## Supporting information




**Figure S1**. Population structure of 750 soybean accessions.
**Figure S2**. Genome‐wide selective sweeps during domestication and modern breeding.
**Table S1**. Accession distribution of various germplasm types in ecoregions.
**Table S2**. Distribution of SNP markers on chromosomes.
**Table S3**. Summary of SNPLDB markers on chromosomes.
**Table S4**. Frequency distribution of allele number.
**Table S5**. The successive allele changes from WA to LR and then to RC.
**Table S6**. The locus polymorphism disappeared and emerged (or locus‐zero/one change) during WA→LR→RC.
**Table S7**. The locus number with allele zero/one or ordinary frequency changes during WA→LR→RC.
**Table S8**. Putative regions experiencing domestication selective sweeps.
**Table S9**. Putative regions experiencing modern breeding selective sweeps.
**Table S10**. The frequency distribution of alleles from WA_SC_ to WA_NC_.
**Table S11**. The frequency distribution of alleles from WA_NC_ to WA_NEC_.
**Table S12**. The frequency distribution of alleles from LR_SC_ to LR_NC_.
**Table S13**. The frequency distribution of alleles from LR_NC_ to LR_NEC_.
**Table S14**. The frequency distribution of alleles from LR_SC_ to RC_SC_.
**Table S15**. The frequency distribution of alleles from LR_NC_ to RC_NC_.
**Table S16**. The frequency distribution of alleles from LR_NEC_ to RC_NEC_.
**Table S17**. Soybean seed weight/size genes identified by selective sweeps and GLAC in domestication and modern breeding (LR vs. WA and RC vs. LR).

**Video S1**.

## Data Availability

The SNPLDBs and the codes have been deposited into GitHub database (https://github.com/njau‐sri/huxinyang‐glac).
