# Supplementary material for: Genome‐wide locus–allele comparison reveals differential evolution dynamics from annual wild to landrace and released cultivar soybeans
Source: Plant Genome. 2025 May 14;18(2):e70037. doi: 10.1002/tpg2.70037 (PMC12078872; doi:10.1002/tpg2.70037)
Supplement: Supplementary file 1 — Figure S1. Population structure of 750 soybean accessions. Figure S2. Genome‐wide selective sweeps during domestication and modern breeding. Table S1. Accession distribution of various germplasm types in ecoregions. Table S2. Distribution of SNP markers on chromosomes. Table S3. Summary of SNPLDB markers on chromosomes. Table S4. Frequency distribution of allele number. Table S5. The successive allele changes from WA to LR and then to RC. Table S6. The locus polymorphism disappeared and emerged (or locus‐zero/one change) during WA→LR→RC. Table S7. The locus number with allele zero/one or ordinary frequency changes during WA→LR→RC. Table S8. Putative regions experiencing domestication selective sweeps. Table S9. Putative regions experiencing modern breeding selective sweeps. Table S10. The frequency distribution of alleles from WASC to WANC. Table S11. The frequency distribution of alleles from WANC to WANEC. Table S12. The frequency distribution of alleles from LRSC to LRNC. Table S13. The frequency distribution of alleles from LRNC to LRNEC. Table S14. The frequency distribution of alleles from LRSC to RCSC. Table S15. The frequency distribution of alleles from LRNC to RCNC. Table S16. The frequency distribution of alleles from LRNEC to RCNEC. Table S17. Soybean seed weight/size genes identified by selective sweeps and GLAC in domestication and modern breeding (LR vs. WA and RC vs. LR). [file TPG2-18-e70037-s002.docx]

**Figure S1. Population structure of 750 soybean accessions.**

(a), (b) and (c) PCA plots of the first three components of 750 accessions using whole-genome SNPLDBs. (d) Phylogenetic tree of all accessions inferred from whole-genome SNPLDBs. The alphabets on the branch indicate the group name of each clade. WA, wild accession; LR, landrace; RC, released cultivar; SC, southern China; NC, northern China; NEC, northeastern China; WA_SC_, wild accession in southern China, and the similar for others. The cultivated soybeans forming a tight cluster that is clearly separate from wild soybeans, and the cultivars show NEC, NC and SC geographic distribution patterns.

In (d), many LRs were closed to RCs and a few WAs located in the LR clades. This is because the country-wide exchange of materials due to natural and artificial reasons which causing germplasm inter-infiltration in a long term. Therefore, among the groups of WA, LR, and RC, there are variation within each group, even with overlaps between WA and LR and between LR and RC, especially the later because of related pedigree, short breeding history and inter-infiltration among eco-regions. In addition, there appeared a few wild accession(s) with vining growth, black seeds but larger seeds and leave areas collected from wild field.

**Figure S2. Genome-wide selective sweeps during domestication and modern breeding.**

(a) and (b) show the whole-genome selective sweeps during domestication and modern breeding, respectively. The XP-CLR values are plotted against the position on each of the 20 chromosomes. The green horizontal solid lines indicate the genome-wide threshold (5% cutoff level) of selective sweeps. For domestication (a), the threshold is XP-CLR ≥ 128.83, and for modern breeding (b), the threshold is XP-CLR ≥ 8.03.

**Table S1. Accession distribution of various germplasm types in ecoregions.**

| **Germplasm type** | **Ecoregion** | | | **Total** |
| --- | --- | --- | --- | --- |
|  | **NEC** | **NC** | **SC** |  |
| **WA** | 27 | 37 | 63 | 127 |
| **LR** | 29 | 67 | 328 | 424 |
| **RC** | 48 | 67 | 84 | 199 |
| **Total** | 104 | 171 | 475 | 750 |

NEC, northeastern China; NC, northern China; SC, southern China; WA, wild accession; LR, landrace; RC, released cultivar.

**Table S2. Distribution of SNP markers on chromosomes.**

| **Chr.** | **No.** | **Min.** | **Max.** | **Mean** |
| --- | --- | --- | --- | --- |
|  |  | **(bp)** | **(kb)** | **(bp)** |
| Gm01 | 154,260 | 1 | 212.8 | 355.4 |
| Gm02 | 126,737 | 1 | 253.7 | 383.2 |
| Gm03 | 149,458 | 1 | 374.1 | 306.2 |
| Gm04 | 168,715 | 1 | 202.0 | 310.5 |
| Gm05 | 99,640 | 1 | 96.0 | 423.8 |
| Gm06 | 150,824 | 1 | 324.6 | 340.9 |
| Gm07 | 114,611 | 1 | 178.8 | 389.4 |
| Gm08 | 126,442 | 1 | 108.2 | 378.0 |
| Gm09 | 138,882 | 1 | 331.8 | 361.4 |
| Gm10 | 140,340 | 1 | 217.2 | 367.4 |
| Gm11 | 106,938 | 1 | 110.7 | 324.7 |
| Gm12 | 104,622 | 1 | 109.9 | 383.2 |
| Gm13 | 122,115 | 1 | 88.6 | 367.0 |
| Gm14 | 150,334 | 1 | 126.8 | 326.2 |
| Gm15 | 176,441 | 1 | 435.4 | 293.3 |
| Gm16 | 118,264 | 1 | 332.0 | 320.3 |
| Gm17 | 112,236 | 1 | 162.7 | 371.0 |
| Gm18 | 194,807 | 1 | 374.3 | 297.8 |
| Gm19 | 156,634 | 1 | 355.5 | 324.0 |
| Gm20 | 133,337 | 1 | 158.5 | 314.2 |
| **Total** | **2,745,637** | **1** | **435.4** | **342.3** |

Chr., chromosome; No., the number of SNPs; Min., Max. and Mean, minimum, maximum and average distance between adjacent markers, respectively.

**Table S3. Summary of SNPLDB markers on chromosomes.**

| **Chr.** | **SNPLDB No.** | **Allele No.** | **Min.** | **Max.** | **Allele/SNPLDB** |
| --- | --- | --- | --- | --- | --- |
| Gm01 | 7,010 | 25,060 | 2 | 22 | 3.6 |
| Gm02 | 7,508 | 27,571 | 2 | 24 | 3.7 |
| Gm03 | 7,246 | 27,399 | 2 | 21 | 3.8 |
| Gm04 | 7,939 | 29,209 | 2 | 26 | 3.7 |
| Gm05 | 7,952 | 27,108 | 2 | 20 | 3.4 |
| Gm06 | 8,632 | 31,616 | 2 | 21 | 3.7 |
| Gm07 | 8,358 | 29,674 | 2 | 23 | 3.6 |
| Gm08 | 9,738 | 33,551 | 2 | 21 | 3.4 |
| Gm09 | 6,382 | 24,139 | 2 | 27 | 3.8 |
| Gm10 | 7,761 | 27,225 | 2 | 20 | 3.5 |
| Gm11 | 6,402 | 22,588 | 2 | 20 | 3.5 |
| Gm12 | 8,408 | 26,937 | 2 | 20 | 3.2 |
| Gm13 | 10,088 | 38,383 | 2 | 24 | 3.8 |
| Gm14 | 7,200 | 25,383 | 2 | 22 | 3.5 |
| Gm15 | 7,275 | 26,783 | 2 | 20 | 3.7 |
| Gm16 | 6,617 | 27,536 | 2 | 23 | 4.2 |
| Gm17 | 6,624 | 23,709 | 2 | 21 | 3.6 |
| Gm18 | 8,440 | 34,914 | 2 | 21 | 4.1 |
| Gm19 | 8,453 | 28,208 | 2 | 18 | 3.3 |
| Gm20 | 6,055 | 22,618 | 2 | 22 | 3.7 |
| **Total** | **154,088** | **559,611** | **2** | **27** | **3.6** |

Chr., chromosome; SNPLDB No., the number of SNPLDBs; Allele No., the number of alleles; Min., Max. and Allele/SNPLDB, minimum, maximum and average allele numbers in SNPLDBs, respectively.

**Table S4. Frequency distribution of allele number.**

| **Allele No.** | **SNPLDB No.** | |
| --- | --- | --- |
| 2 | 83058 | 53.90% |
| 3 | 22593 | 14.66% |
| 4 | 10697 | 6.94% |
| 5 | 8727 | 5.66% |
| 6 | 8067 | 5.24% |
| 7 | 6473 | 4.20% |
| 8 | 4820 | 3.13% |
| 9 | 3256 | 2.11% |
| 10 | 2192 | 1.42% |
| 11 | 1298 | 0.84% |
| 12 | 831 | 0.54% |
| 13 | 603 | 0.39% |
| 14 | 425 | 0.28% |
| 15 | 320 | 0.21% |
| 16 | 235 | 0.15% |
| 17 | 164 | 0.11% |
| 18 | 121 | 0.08% |
| 19 | 77 | 0.05% |
| 20 | 56 | 0.04% |
| 21 | 37 | 0.02% |
| 22 | 18 | 0.01% |
| 23 | 14 | 0.01% |
| 24 | 2 | 0.00% |
| 25 | 1 | 0.00% |
| 26 | 1 | 0.00% |
| 27 | 2 | 0.00% |

Allele No., the number of alleles; SNPLDB No., the number of SNPLDBs.

**Table S5. The successive allele changes from WA to LR and then to RC.**

| Allele type | Allele  number | % in  total | % in  item |
| --- | --- | --- | --- |
| 1. LR vs. WA excluded | 76,866 | 13.74 |  |
| RC vs. LR frequency of 0 | **70,628** | **12.62** | **91.88** |
| RC vs. LR inherited from WA as direct parent in RC | 6,238 | 1.11 | 8.12 |
| 2. LR vs. WA emerged | 37,543 | 6.71 |  |
| RC vs. LR excluded | 1,853 | 0.33 | 4.94 |
| RC vs. LR ordinary frequency change | **35,690** | **6.38** | **95.06** |
| 3. LR vs. WA frequency increase to 1 (LPD) | 23,670 | 4.23 |  |
| RC vs. LR frequency of 1 | **22,261** | **3.98** | **94.05** |
| RC vs. LR frequency decrease from 1 (LPR) | 1,409 | 0.25 | 5.95 |
| 4. LR vs. WA frequency decrease from 1 (LPE) | 2,351 | 0.42 |  |
| RC vs. LR frequency increase to 1 (LPD) | 130 | 0.02 | 5.53 |
| RC vs. LR ordinary frequency change | 2,221 | 0.40 | 94.47 |
| 5. LR vs. WA ordinary frequency increase | 206,020 | 36.81 |  |
| RC vs. LR excluded | 2,274 | 0.41 | 1.10 |
| RC vs. LR frequency increase to 1 (LPD) | **23,571** | **4.21** | **11.44** |
| RC vs. LR ordinary frequency increase | 83,596 | 14.94 | 40.58 |
| RC vs. LR ordinary frequency decrease | 96,579 | 17.26 | 46.88 |
| 6. LR vs. WA ordinary frequency decrease | 212,521 | 37.98 |  |
| RC vs. LR excluded | **75,446** | **13.48** | **35.50** |
| RC vs. LR frequency increase to 1 (LPD) | 270 | 0.05 | 0.13 |
| RC vs. LR ordinary frequency increase | 70,143 | 12.53 | 33.01 |
| RC vs. LR ordinary frequency decrease | 66,662 | 11.91 | 31.37 |
| 7. LR vs. WA frequency of 0 then RC vs. LR emerged | 603 | 0.11 |  |
| 8. LR vs. WA frequency of 1 then RC vs. LR frequency decrease from 1 (LPE) | 37 | 0.01 |  |

“% in total” means the corresponding alleles accounting for the total 559,611 alleles, while “% in item” means the corresponding alleles accounting for that of the allele type. LPD, LPE and LPR mean locus polymorphism disappeared, emerged and restored.

Boldface signifies the important outcomes.

**Table S6. The locus polymorphism disappeared and emerged (or locus-zero/one change) during WA→LR→RC.**

| Locus type | Locus number | % |
| --- | --- | --- |
| WA fixed locus | 2,388 | **1.55** |
| LR fixed locus | 23,707 | **15.39** |
| RC fixed locus | 46,232 | **30.00** |
| LR vs. WA, increase to 1 (LPD) | 23,670 | 15.36 |
| RC vs. LR, frequency of 1 | 22,261 | **14.45** |
| RC vs. LR, decrease from 1 (LPR) | 1,409 | 0.91 |
| LR vs. WA, decrease from 1 (LPE) | 2,351 | 1.53 |
| RC vs. LR, increase to 1 (LPD) | 130 | 0.08 |
| RC vs. LR, ordinary frequency increase | 946 | 0.61 |
| RC vs. LR, ordinary frequency decrease | 1,275 | 0.83 |
| LR vs. WA, ordinary frequency increase and RC vs. LR, increase to 1 (LPD) | 23,571 | **15.30** |
| LR frequency <0.9 | 2 | 0.00 |
| LR frequency ≥ 0.9 and < 0.95 | 175 | 0.11 |
| LR frequency ≥ 0.95 | 23,394 | **15.18** |
| LR vs. WA, ordinary frequency decrease and RC vs. LR, increase to 1 (LPD) | 270 | 0.18 |
| LR vs. WA, frequency of 1 and RC vs. LR decrease from 1 (LPE) | 37 | 0.02 |

“%”: the percentage of the respective locus number accounting for the total locus number (154,088) in whole genome. Fixed locus: the fixation locus which only have an allele frequency of 1. LPD, LPE and LPR mean locus polymorphism disappeared, emerged and restored. There were 23,670 (15.36%) loci disappeared and 2,351 (1.53%) loci emerged during domestication, and 130 (0.08%) plus 23,571 (15.30%) plus 270 (0.18%) in a total of 23,971 (15.56%) loci disappeared and 37 (0.02%) loci emerged during modern breeding. From WA to LR and then to RC, the disappeared locus number was more than the emerged locus number, which caused the fixed locus number continuously increasing from 2,388 (1.55%) to 23,707 (15.39%) then to 46,232 (30.00%).

Boldface signifies the important outcomes.

**Table S7. The locus number with allele-zero/one or ordinary frequency changes during WA→LR→RC.**

| **Item** | **LR vs. WA** | **RC vs. LR** |
| --- | --- | --- |
| With allele-zero/one changes | 70,363 (45.66%) | 61,308 (39.79%) |
| With ordinary frequency changes | 128,030 (83.09%) | 106,410 (69.06%) |
| With both allele-zero/one and ordinary frequency changes | 44,342 (28.78%) | 37,299 (24.21%) |
| With only allele-zero/one changes | 26,021 (16.89%) | 24,009 (15.58%) |
| With only ordinary frequency changes | 83,688 (54.31%) | 69,111 (44.85%) |
| With only allele frequency of 1 | 37 (0.02%) | 22,261 (14.45%) |

The respective percentage is relative to the total locus number (154,088) in whole genome.

**Table S8. Putative regions experiencing domestication selective sweeps.**

| **Chr.** | **Start** | **End** | **Max XP-CLR** |
| --- | --- | --- | --- |
| 1 | 23,630,001 | 25,440,000 | 608.9 |
| 1 | 26,420,001 | 27,080,000 | 760.58 |
| 1 | 35,680,001 | 36,130,000 | 658.35 |
| 1 | 36,150,001 | 36,540,000 | 333.55 |
| 1 | 37,010,001 | 37,340,000 | 236.59 |
| 1 | 46,010,001 | 46,770,000 | 700.24 |
| 1 | 47,500,001 | 48,280,000 | 897.08 |
| 2 | 38,190,001 | 38,520,000 | 549.11 |
| 3 | 18,290,001 | 18,640,000 | 349.86 |
| 3 | 31,310,001 | 31,980,000 | 471.93 |
| 3 | 32,090,001 | 32,690,000 | 574.96 |
| 3 | 36,940,001 | 37,360,000 | 512.7 |
| 3 | 40,220,001 | 40,920,000 | 676.2 |
| 4 | 600,001 | 970,000 | 416.43 |
| 4 | 7,390,001 | 7,950,000 | 323.88 |
| 4 | 44,740,001 | 45,260,000 | 506.17 |
| 4 | 45,420,001 | 45,920,000 | 630.94 |
| 5 (5-1) | 1,480,001 | 2,100,000 | 472.72 |
| 5 | 5,740,001 | 6,780,000 | 749.3 |
| 5 | 6,810,001 | 7,500,000 | 673 |
| 5 | 9,170,001 | 10,190,000 | 423.93 |
| 5 | 11,250,001 | 11,600,000 | 240.63 |
| 5 | 12,350,001 | 12,670,000 | 240.15 |
| 5 | 15,630,001 | 16,040,000 | 190.48 |
| 5 | 23,110,001 | 23,440,000 | 194.49 |
| 5 | 29,700,001 | 30,370,000 | 460.47 |
| 5 | 30,650,001 | 31,000,000 | 737.88 |
| 6 | 1,600,001 | 2,210,000 | 357.7 |
| 6 | 7,070,001 | 7,550,000 | 438.75 |
| 6 | 9,210,001 | 10,220,000 | 796.07 |
| 6 | 10,370,001 | 10,670,000 | 325.27 |
| 7 | 14,510,001 | 14,870,000 | 530.74 |
| 7 | 17,420,001 | 17,720,000 | 667.36 |
| 7 | 36,060,001 | 36,390,000 | 362.74 |
| 7 | 39,200,001 | 40,100,000 | 665.14 |
| 7 | 40,620,001 | 41,820,000 | 574.63 |
| 7 | 41,850,001 | 42,290,000 | 674.15 |
| 7 | 43,180,001 | 43,660,000 | 487.36 |
| 7 | 43,740,001 | 44,230,000 | 772.82 |
| 8 | 1 | 640,000 | 490.89 |
| 8 | 780,001 | 1,160,000 | 454.56 |
| 8 | 3,610,001 | 4,520,000 | 850.84 |
| 8 | 4,530,001 | 4,850,000 | 275.83 |
| 8 | 5,740,001 | 6,920,000 | 576.23 |
| 8 | 21,310,001 | 21,830,000 | 577.06 |
| 8 | 23,040,001 | 23,350,000 | 702.42 |
| 8 | 24,860,001 | 25,400,000 | 420.55 |
| 8 | 25,470,001 | 25,800,000 | 263.24 |
| 8 | 26,090,001 | 26,430,000 | 287.62 |
| 8 | 27,000,001 | 27,360,000 | 275.91 |
| 8 | 27,730,001 | 28,140,000 | 283.55 |
| 8 | 30,500,001 | 30,850,000 | 232.15 |
| 8 | 30,940,001 | 31,240,000 | 183.34 |
| 8 | 31,490,001 | 32,040,000 | 322.81 |
| 8 | 32,750,001 | 33,610,000 | 240.81 |
| 8 | 35,850,001 | 36,160,000 | 591.94 |
| 8 | 44,170,001 | 44,590,000 | 431.81 |
| 9 | 32,980,001 | 34,350,000 | 510.97 |
| 9 | 34,640,001 | 35,010,000 | 278.27 |
| 10 | 6,690,001 | 7,280,000 | 377.69 |
| 10 | 11,410,001 | 11,860,000 | 318.91 |
| 10 | 37,310,001 | 38,260,000 | 663.5 |
| 10 | 40,800,001 | 41,140,000 | 416.51 |
| 10 | 45,290,001 | 46,230,000 | 631.45 |
| 11 | 2,250,001 | 2,560,000 | 512.81 |
| 11 | 2,790,001 | 3,230,000 | 588.36 |
| 11 | 9,440,001 | 9,970,000 | 591.58 |
| 11 | 12,000,001 | 12,590,000 | 585.76 |
| 11 | 26,560,001 | 27,110,000 | 652.43 |
| 11 | 31,190,001 | 31,640,000 | 457.53 |
| 11 | 32,770,001 | 33,170,000 | 498.24 |
| 12 | 3,220,001 | 3,560,000 | 374.29 |
| 12 | 3,570,001 | 3,920,000 | 466.54 |
| 12 | 4,710,001 | 5,340,000 | 635.12 |
| 12 | 6,990,001 | 7,780,000 | 598.76 |
| 12 | 12,470,001 | 13,010,000 | 429.46 |
| 12 | 13,020,001 | 13,580,000 | 384.01 |
| 12 | 13,880,001 | 14,240,000 | 293.19 |
| 12 | 14,920,001 | 15,470,000 | 478.31 |
| 12 | 17,290,001 | 17,610,000 | 350.24 |
| 12 | 18,540,001 | 19,220,000 | 446.31 |
| 12 | 19,410,001 | 20,500,000 | 385.71 |
| 12 | 21,710,001 | 22,020,000 | 246.09 |
| 12 | 23,280,001 | 23,710,000 | 336.4 |
| 12 | 31,830,001 | 32,370,000 | 890.23 |
| 12 | 32,740,001 | 33,040,000 | 613.65 |
| 12 | 33,810,001 | 34,370,000 | 673.62 |
| 13 | 19,650,001 | 20,130,000 | 453.87 |
| 13 | 20,990,001 | 21,500,000 | 533.36 |
| 13 | 22,540,001 | 23,060,000 | 510.51 |
| 13 | 26,200,001 | 26,680,000 | 381.05 |
| 13 | 27,110,001 | 27,430,000 | 316.43 |
| 13 | 34,840,001 | 35,340,000 | 816.97 |
| 13 | 38,610,001 | 39,170,000 | 686.68 |
| 13 | 45,060,001 | 45,370,000 | 364.29 |
| 14 | 13,560,001 | 13,970,000 | 374.81 |
| 14 | 14,020,001 | 14,400,000 | 431.09 |
| 14 | 21,310,001 | 21,690,000 | 369.11 |
| 14 | 44,770,001 | 45,450,000 | 1,009.10 |
| 15 | 11,200,001 | 11,530,000 | 697.69 |
| 15 | 49,120,001 | 49,540,000 | 382.2 |
| 16 | 1,880,001 | 2,270,000 | 447.08 |
| 17 | 6,580,001 | 7,060,000 | 683.06 |
| 17 | 9,380,001 | 9,940,000 | 784.29 |
| 17 | 10,250,001 | 10,820,000 | 382.18 |
| 17 | 39,530,001 | 39,840,000 | 180.84 |
| 18 (18-1) | 5,480,001 | 6,060,000 | 913.49 |
| 18 | 9,410,001 | 9,710,000 | 294.09 |
| 19 | 4,330,001 | 5,120,000 | 678.58 |
| 19 | 28,130,001 | 28,850,000 | 405.02 |
| 19 | 29,080,001 | 29,380,000 | 200.21 |
| 19 | 29,780,001 | 30,200,000 | 589.77 |
| 19 | 31,240,001 | 31,720,000 | 575.81 |
| 19 | 32,500,001 | 32,840,000 | 405.69 |
| 19 | 32,860,001 | 33,330,000 | 312.38 |
| 19 | 33,530,001 | 33,900,000 | 335.04 |
| 19 | 38,780,001 | 39,090,000 | 323.95 |
| 19 | 41,630,001 | 42,240,000 | 518.36 |
| 19 | 42,530,001 | 42,870,000 | 380.76 |
| 19 | 43,240,001 | 44,500,000 | 771.71 |
| 19 | 46,180,001 | 46,920,000 | 732.96 |
| 20 | 4,810,001 | 5,120,000 | 624.71 |
| 20 | 6,610,001 | 7,030,000 | 383.5 |
| 20 | 7,510,001 | 7,970,000 | 316.6 |
| 20 | 8,000,001 | 8,810,000 | 846.09 |
| 20 | 8,850,001 | 10,890,000 | 1,160.46 |
| 20 | 10,930,001 | 12,080,000 | 924.55 |
| 20 | 16,640,001 | 17,340,000 | 411.7 |
| 20 | 17,960,001 | 18,300,000 | 285.93 |
| 20 | 18,310,001 | 19,690,000 | 669.24 |
| 20 | 20,160,001 | 21,090,000 | 489.81 |
| 20 | 21,140,001 | 21,980,000 | 402.52 |
| 20 | 22,290,001 | 22,790,000 | 479.04 |
| 20 | 22,870,001 | 23,600,000 | 329.39 |
| 20 | 24,140,001 | 24,620,000 | 654.28 |
| 20 | 24,680,001 | 25,090,000 | 316.54 |
| 20 | 25,130,001 | 25,530,000 | 352.54 |
| 20 | 31,960,001 | 32,700,000 | 421.42 |
| 20 | 32,830,001 | 33,650,000 | 652.19 |
| 20 | 39,980,001 | 40,490,000 | 469.09 |

The threshold is XP-CLR ≥ 128.83 for domestication.

See Table S17 for the location of domestication selective sweeps, like (5-1).

**Table S9. Putative regions experiencing modern breeding selective sweeps.**

| **Chr.** | **Start** | **End** | **Max XP-CLR** |
| --- | --- | --- | --- |
| 1 [1-1] | 8,170,001 | 8,480,000 | 14.67 |
| 1 | 23,840,001 | 24,150,000 | 12.14 |
| 1 | 24,670,001 | 25,020,000 | 9.82 |
| 1 | 26,030,001 | 26,350,000 | 50.13 |
| 1 | 26,540,001 | 26,910,000 | 35.26 |
| 1 | 35,670,001 | 36,020,000 | 17.18 |
| 1 | 36,390,001 | 36,710,000 | 16.37 |
| 1 | 53,120,001 | 53,540,000 | 71.85 |
| 2 | 1,180,001 | 1,670,000 | 73.02 |
| 2 | 1,730,001 | 2,180,000 | 34.12 |
| 2 | 13,020,001 | 13,450,000 | 51.03 |
| 2 | 15,140,001 | 15,450,000 | 69.01 |
| 2 | 28,000,001 | 28,450,000 | 19.54 |
| 2 | 28,740,001 | 29,230,000 | 31.08 |
| 2 | 30,160,001 | 30,580,000 | 18.07 |
| 2 | 31,080,001 | 31,500,000 | 20.28 |
| 2 | 32,030,001 | 32,410,000 | 21.98 |
| 2 | 32,530,001 | 32,860,000 | 22.01 |
| 2 | 33,590,001 | 33,890,000 | 23.82 |
| 2 | 34,480,001 | 35,250,000 | 28.39 |
| 2 [2-1] | 35,270,001 | 35,860,000 | 23.65 |
| 2 | 36,070,001 | 36,460,000 | 49.02 |
| 2 | 41,760,001 | 42,080,000 | 30.81 |
| 3 | 31,310,001 | 31,670,000 | 77.96 |
| 3 | 31,970,001 | 32,280,000 | 112.11 |
| 4 | 1,190,001 | 1,550,000 | 106.07 |
| 4 | 6,060,001 | 6,390,000 | 40.51 |
| 4 | 26,160,001 | 26,490,000 | 30.07 |
| 4 | 28,570,001 | 28,940,000 | 35.99 |
| 4 | 31,300,001 | 31,610,000 | 39.72 |
| 4 | 31,650,001 | 32,000,000 | 41.19 |
| 4 | 34,060,001 | 34,500,000 | 20.84 |
| 4 | 35,610,001 | 36,000,000 | 32.75 |
| 4 | 37,530,001 | 37,980,000 | 53.54 |
| 4 | 38,980,001 | 39,480,000 | 54.8 |
| 4 | 50,270,001 | 50,760,000 | 56.12 |
| 4 | 51,980,001 | 52,450,000 | 59.71 |
| 5 | 920,001 | 1,220,000 | 36.96 |
| 5 [5-1] | 1,660,001 | 1,990,000 | 71.33 |
| 5 | 4,130,001 | 4,520,000 | 31.64 |
| 5 | 26,100,001 | 26,410,000 | 63.49 |
| 5 | 29,210,001 | 29,600,000 | 33.83 |
| 5 | 29,770,001 | 31,140,000 | 80.13 |
| 6 | 9,600,001 | 10,100,000 | 44.18 |
| 6 | 29,450,001 | 29,780,000 | 43.31 |
| 6 | 30,580,001 | 30,910,000 | 12.16 |
| 6 | 35,020,001 | 36,150,000 | 44.98 |
| 6 | 36,160,001 | 36,660,000 | 20.73 |
| 6 | 36,690,001 | 37,020,000 | 17.6 |
| 6 | 43,090,001 | 43,450,000 | 17.03 |
| 6 | 44,700,001 | 45,000,000 | 29.26 |
| 6 | 45,560,001 | 45,930,000 | 18.6 |
| 7 | 16,220,001 | 16,640,000 | 63.67 |
| 7 | 40,700,001 | 41,210,000 | 232.81 |
| 8 | 3,860,001 | 4,310,000 | 72.28 |
| 8 | 5,710,001 | 6,550,000 | 353.23 |
| 8 | 6,620,001 | 7,100,000 | 92.38 |
| 8 | 7,920,001 | 8,320,000 | 99.06 |
| 8 | 8,920,001 | 9,270,000 | 34.68 |
| 8 | 12,940,001 | 13,300,000 | 26.83 |
| 8 | 13,680,001 | 14,100,000 | 179.48 |
| 8 | 15,160,001 | 15,460,000 | 79.31 |
| 8 | 17,080,001 | 17,480,000 | 75.7 |
| 8 | 20,170,001 | 20,770,000 | 63.68 |
| 8 | 38,800,001 | 39,320,000 | 26.39 |
| 8 | 39,340,001 | 40,280,000 | 97.21 |
| 8 | 40,890,001 | 41,610,000 | 73.76 |
| 9 | 2,410,001 | 2,730,000 | 35.62 |
| 9 | 35,230,001 | 35,610,000 | 26.27 |
| 9 | 42,540,001 | 42,870,000 | 46.29 |
| 9 | 44,480,001 | 44,870,000 | 301.39 |
| 10 | 37,630,001 | 38,110,000 | 191.59 |
| 10 | 42,200,001 | 42,530,000 | 18.22 |
| 10 | 43,910,001 | 44,210,000 | 52.67 |
| 10 | 47,380,001 | 47,880,000 | 86.86 |
| 11 | 2,770,001 | 3,460,000 | 70.78 |
| 11 | 8,580,001 | 8,890,000 | 36.2 |
| 11 | 10,450,001 | 10,830,000 | 40.8 |
| 11 | 13,470,001 | 14,020,000 | 27.44 |
| 11 | 14,090,001 | 14,680,000 | 107.84 |
| 11 | 25,930,001 | 26,280,000 | 46.31 |
| 11 | 26,570,001 | 26,880,000 | 80.47 |
| 12 | 4,980,001 | 5,710,000 | 164.4 |
| 12 | 7,600,001 | 7,960,000 | 119.33 |
| 12 | 18,470,001 | 19,340,000 | 60.11 |
| 12 | 20,100,001 | 20,430,000 | 30.25 |
| 12 | 21,120,001 | 22,440,000 | 156.8 |
| 12 | 22,910,001 | 24,050,000 | 184.26 |
| 12 | 24,670,001 | 25,010,000 | 103.74 |
| 12 | 25,050,001 | 25,630,000 | 68.72 |
| 12 | 25,670,001 | 26,060,000 | 33.19 |
| 12 | 26,770,001 | 27,610,000 | 150.27 |
| 12 | 27,630,001 | 28,210,000 | 29.86 |
| 12 | 28,270,001 | 28,640,000 | 30.46 |
| 12 | 28,870,001 | 29,800,000 | 119.78 |
| 12 | 29,810,001 | 30,250,000 | 37.5 |
| 12 | 30,260,001 | 30,560,000 | 20.72 |
| 12 | 30,580,001 | 31,260,000 | 37.77 |
| 12 | 31,270,001 | 32,540,000 | 223.73 |
| 12 | 32,620,001 | 33,030,000 | 137.53 |
| 12 | 36,420,001 | 36,960,000 | 35.07 |
| 13 | 17,650,001 | 18,000,000 | 41.02 |
| 13 | 18,460,001 | 18,770,000 | 92.14 |
| 13 | 19,590,001 | 20,140,000 | 119.68 |
| 13 | 20,610,001 | 20,930,000 | 45.2 |
| 13 | 29,010,001 | 29,690,000 | 51.67 |
| 13 | 34,930,001 | 35,320,000 | 37.62 |
| 13 | 40,110,001 | 40,510,000 | 40.42 |
| 13 | 43,560,001 | 44,120,000 | 130.87 |
| 13 | 45,500,001 | 45,970,000 | 62.18 |
| 14 | 14,290,001 | 14,670,000 | 16.23 |
| 14 | 19,870,001 | 20,420,000 | 146.22 |
| 14 | 21,060,001 | 22,540,000 | 175.31 |
| 14 | 23,300,001 | 23,620,000 | 35.89 |
| 14 | 30,530,001 | 30,880,000 | 22.04 |
| 14 | 40,540,001 | 41,360,000 | 50.56 |
| 14 | 41,620,001 | 41,940,000 | 25.38 |
| 14 | 44,030,001 | 44,440,000 | 107.42 |
| 14 | 44,810,001 | 45,490,000 | 60.64 |
| 15 | 410,001 | 890,000 | 35.02 |
| 15 | 1,050,001 | 1,520,000 | 96.5 |
| 15 [15-1] | 3,620,001 | 4,130,000 | 152.12 |
| 15 | 12,140,001 | 12,490,000 | 72.82 |
| 15 | 45,310,001 | 45,800,000 | 49.31 |
| 16 | 1,940,001 | 2,310,000 | 90.59 |
| 16 | 7,010,001 | 7,450,000 | 63.75 |
| 16 | 29,890,001 | 30,230,000 | 17.16 |
| 17 | 1,100,001 | 1,440,000 | 66.63 |
| 17 | 1,460,001 | 1,810,000 | 49.4 |
| 17 [17-1] | 2,440,001 | 3,060,000 | 125.48 |
| 17 | 5,950,001 | 6,320,000 | 40.37 |
| 17 | 6,350,001 | 6,760,000 | 572.58 |
| 18 | 38,460,001 | 39,290,000 | 150.2 |
| 18 | 39,770,001 | 40,320,000 | 110.89 |
| 18 | 40,360,001 | 41,260,000 | 88.17 |
| 18 | 45,140,001 | 45,700,000 | 332.65 |
| 18 | 45,760,001 | 46,810,000 | 394.25 |
| 19 | 2,510,001 | 2,920,000 | 45.27 |
| 19 | 4,330,001 | 4,670,000 | 53.52 |
| 19 [19-1] | 5,260,001 | 6,090,000 | 49.51 |
| 19 | 39,110,001 | 39,420,000 | 128.11 |
| 19 | 42,800,001 | 43,220,000 | 29.03 |
| 19 | 43,970,001 | 44,340,000 | 28.28 |
| 20 | 4,940,001 | 5,650,000 | 47.44 |
| 20 | 5,670,001 | 6,350,000 | 72.47 |
| 20 | 6,370,001 | 7,420,000 | 136.15 |
| 20 | 7,430,001 | 8,670,000 | 159.77 |
| 20 | 9,550,001 | 10,030,000 | 38.88 |
| 20 | 10,400,001 | 10,830,000 | 33.91 |
| 20 | 33,210,001 | 33,670,000 | 125.72 |

The threshold is XP-CLR ≥ 8.03 for modern breeding.

See Table S17 for the location of modern breeding selective sweeps, like [1-1].

**Table S10. The frequency distribution of alleles from WA_SC_ to WA_NC_.**

| **Allele number** | | **Class limit of allele frequency in WA_SC_** | | | | | | | | | | | |
| --- | --- | --- | --- | --- | --- | --- | --- | --- | --- | --- | --- | --- | --- |
|  |  | **0** | **0.0-0.1** | **0.1-0.2** | **0.2-0.3** | **0.3-0.4** | **0.4-0.5** | **0.5-0.6** | **0.6-0.7** | **0.7-0.8** | **0.8-0.9** | **0.9-0.99** | **1** |
| **Class limit of allele frequency in WA_NC_** | **0** | 46,397 | **34,724** | **5,523** | **1,001** | **132** | **19** | **2** |  |  |  |  |  |
|  | **0.0-0.1** | **11,583** | 59,944 | 32,575 | 10,848 | 3,109 | 528 | 101 | 9 |  |  |  |  |
|  | **0.1-0.2** | **1,525** | 35,400 | 34,413 | 20,405 | 10,103 | 3,061 | 943 | 221 | 18 |  |  |  |
|  | **0.2-0.3** | **214** | 13,109 | 16,771 | 14,536 | 10,887 | 4,979 | 2,418 | 918 | 194 | 21 |  |  |
|  | **0.3-0.4** | **13** | 5,104 | 6,487 | 6,226 | 6,248 | 4,081 | 2,769 | 1,600 | 475 | 124 | 9 |  |
|  | **0.4-0.5** |  | 2,775 | 5,102 | 4,984 | 5,930 | 4,661 | 3,730 | 3,140 | 1,519 | 644 | 117 |  |
|  | **0.5-0.6** |  | 616 | 1,942 | 2,625 | 3,675 | 3,508 | 3,678 | 3,865 | 2,449 | 1,785 | 593 |  |
|  | **0.6-0.7** |  | 46 | 371 | 670 | 1,474 | 1,864 | 2,332 | 2,986 | 2,450 | 2,177 | 1,277 | **3** |
|  | **0.7-0.8** |  |  | 46 | 218 | 719 | 1,291 | 2,187 | 3,864 | 4,483 | 4,951 | 3,107 | **37** |
|  | **0.8-0.9** |  |  | 1 | 14 | 109 | 359 | 1,017 | 2,879 | 5,367 | 8,293 | 7,370 | **262** |
|  | **0.9-0.99** |  |  |  |  | 1 | 18 | 157 | 781 | 2,757 | 7,103 | 9,820 | **1,085** |
|  | **1** |  |  |  |  |  |  | **6** | **40** | **243** | **1,305** | **3,773** | 3,193 |

“0” row: “top edge” of the table, excluded alleles (frequency most from 0.0-0.3 in WA_SC_); “0” column: “left edge” of the table, emerged alleles (frequency most from 0.0-0.2 in WA_NC_); “1” row: “bottom edge” of the table, excluded loci (frequency most from 0.8-0.99 in WA_SC_); “1” column: “right edge” of the table, emerged loci (frequency most from 0.9-0.99 in WA_NC_).

Boldface signifies the important outcomes.

**Table S11. The frequency distribution of alleles from WA_NC_ to WA_NEC_.**

| **Allele number** | | **Class limit of allele frequency in WA_NC_** | | | | | | | | | | | |
| --- | --- | --- | --- | --- | --- | --- | --- | --- | --- | --- | --- | --- | --- |
|  |  | **0** | **0.0-0.1** | **0.1-0.2** | **0.2-0.3** | **0.3-0.4** | **0.4-0.5** | **0.5-0.6** | **0.6-0.7** | **0.7-0.8** | **0.8-0.9** | **0.9-0.99** | **1** |
| **Class limit of allele frequency in WA_NEC_** | **0** | 61,271 | **31,714** | **7,969** | **904** | **84** | **8** | **2** |  |  |  |  |  |
|  | **0.0-0.1** | **6,979+11,744** | 41,598 | 27,246 | 7,514 | 1,326 | 350 | 40 | 1 |  |  |  |  |
|  | **0.1-0.2** | **1,074+4,912** | 30,519 | 39,171 | 21,468 | 7,141 | 3,804 | 818 | 102 | 20 | 2 |  |  |
|  | **0.2-0.3** | **168+1,309** | 10,821 | 20,167 | 17,384 | 9,857 | 8,067 | 3,318 | 668 | 191 | 15 | 2 |  |
|  | **0.3-0.4** | **24+235** | 2,701 | 6,173 | 7,172 | 5,022 | 4,897 | 3,092 | 1,081 | 437 | 66 | 3 |  |
|  | **0.4-0.5** | **6+67** | 1,133 | 4,158 | 6,309 | 5,228 | 6,389 | 4,954 | 2,246 | 1,412 | 428 | 55 | **0+2** |
|  | **0.5-0.6** | **0+9** | 204 | 1,077 | 2,558 | 2,940 | 4,869 | 4,841 | 3,004 | 2,900 | 1,435 | 330 | **1+19** |
|  | **0.6-0.7** |  | 6 | 114 | 564 | 1,019 | 2,238 | 2,946 | 2,409 | 2,877 | 1,989 | 710 | **3+55** |
|  | **0.7-0.8** |  | 1 | 13 | 164 | 472 | 1,700 | 3,413 | 3,646 | 5,672 | 5,641 | 2,753 | **29+366** |
|  | **0.8-0.9** |  |  | 1 | 10 | 46 | 266 | 1,215 | 2,167 | 5,583 | 9,268 | 6,791 | **177+1,054** |
|  | **0.9-0.99** |  |  |  |  | 1 | 13 | 96 | 308 | 1,620 | 5,409 | 7,068 | **595+1,661** |
|  | **1** |  |  |  |  |  | **1** | **1** | **18** | **191** | **1,418** | **4,010** | 4,598 |

“0” row: “top edge” of the table, excluded alleles (frequency most from 0.0-0.2 in WA_NC_); “0” column: “left edge” of the table, emerged and directly inherited (from WA_SC_) alleles (frequency most from 0.0-0.2 in WA_NEC_); “1” row: “bottom edge” of the table, excluded loci (frequency most from 0.8-0.99 in WA_NC_); “1” column: “right edge” of the table, emerged and restored loci (frequency most from 0.9-0.99 in WA_NEC_).

Boldface signifies the important outcomes.

**Table S12. The frequency distribution of alleles from LR_SC_ to LR_NC_.**

| **Allele number** | | **Class limit of allele frequency in LR_SC_** | | | | | | | | | | | |
| --- | --- | --- | --- | --- | --- | --- | --- | --- | --- | --- | --- | --- | --- |
|  |  | **0** | **0.0-0.1** | **0.1-0.2** | **0.2-0.3** | **0.3-0.4** | **0.4-0.5** | **0.5-0.6** | **0.6-0.7** | **0.7-0.8** | **0.8-0.9** | **0.9-0.99** | **1** |
| **Class limit of allele frequency in LR_NC_** | **0** | 84,097 | **84,365** | **429** | **1** |  |  |  |  |  |  |  |  |
|  | **0.0-0.1** | **1,500+10,477** | 110,176 | 14,992 | 2,370 | 267 | 32 | 1 |  |  |  |  |  |
|  | **0.1-0.2** | **57+483** | 20,993 | 12,945 | 8,261 | 3,321 | 744 | 105 | 9 | 1 |  |  |  |
|  | **0.2-0.3** | **5+54** | 7,863 | 6,134 | 6,269 | 5,459 | 2,960 | 1,058 | 317 | 63 |  |  |  |
|  | **0.3-0.4** | **0+3** | 3,392 | 3,338 | 3,146 | 3,095 | 2,858 | 1,776 | 994 | 280 | 66 |  |  |
|  | **0.4-0.5** |  | 908 | 1,775 | 2,527 | 2,764 | 2,819 | 2,733 | 2,249 | 1,223 | 593 | 30 |  |
|  | **0.5-0.6** |  | 72 | 560 | 1,166 | 1,776 | 2,087 | 2,239 | 2,596 | 2,162 | 1,307 | 453 |  |
|  | **0.6-0.7** |  |  | 35 | 247 | 675 | 1,180 | 1,623 | 2,139 | 2,238 | 2,102 | 1,376 |  |
|  | **0.7-0.8** |  |  | 1 | 53 | 170 | 588 | 1,250 | 2,488 | 2,654 | 2,712 | 2,947 | **0+12** |
|  | **0.8-0.9** |  |  |  |  | 13 | 23 | 245 | 1,086 | 2,446 | 3,860 | 7,425 | **5+151** |
|  | **0.9-0.99** |  |  |  |  |  | 1 | 6 | 41 | 520 | 3,508 | 27,472 | **86+2,720** |
|  | **1** |  |  |  |  |  |  |  |  |  | **65** | **23,276** | 25,377 |

“0” row: “top edge” of the table, excluded alleles (frequency most from 0.0-0.1 in LR_SC_); “0” column: “left edge” of the table, emerged and directly inherited (from WA) alleles (frequency most from 0.0-0.1 in LR_NC_); “1” row: “bottom edge” of the table, excluded loci (frequency most from 0.9-0.99 in LR_SC_); “1” column: “right edge” of the table, emerged and restored loci (frequency most from 0.9-0.99 in LR_NC_).

Boldface signifies the important outcomes.

**Table S13. The frequency distribution of alleles from LR_NC_ to LR_NEC_.**

| **Allele number** | | **Class limit of allele frequency in LR_NC_** | | | | | | | | | | | |
| --- | --- | --- | --- | --- | --- | --- | --- | --- | --- | --- | --- | --- | --- |
|  |  | **0** | **0.0-0.1** | **0.1-0.2** | **0.2-0.3** | **0.3-0.4** | **0.4-0.5** | **0.5-0.6** | **0.6-0.7** | **0.7-0.8** | **0.8-0.9** | **0.9-0.99** | **1** |
| **Class limit of allele frequency in LR_NEC_** | **0** | 142,611 | **53,997** | **2,539** | **210** | **1** |  |  |  |  |  |  |  |
|  | **0.0-0.1** | **701+23,411** | 60,726 | 12,710 | 2,162 | 177 | 9 |  |  |  |  |  |  |
|  | **0.1-0.2** | **73+2,019** | 21,552 | 19,566 | 8,861 | 2,246 | 498 | 65 | 3 |  |  |  |  |
|  | **0.2-0.3** | **0+77** | 3,142 | 8,985 | 9,927 | 5,585 | 2,473 | 578 | 72 | 1 |  |  |  |
|  | **0.3-0.4** |  | 374 | 2,567 | 6,133 | 5,426 | 4,459 | 1,925 | 400 | 60 | 10 |  |  |
|  | **0.4-0.5** |  | 24 | 484 | 2,285 | 3,383 | 4,598 | 2,997 | 1,212 | 398 | 31 |  |  |
|  | **0.5-0.6** |  |  | 61 | 519 | 1,564 | 3,326 | 3,811 | 2,413 | 1,303 | 266 | 9 |  |
|  | **0.6-0.7** |  |  | 7 | 78 | 480 | 1,768 | 3,212 | 3,370 | 3,038 | 1,191 | 102 |  |
|  | **0.7-0.8** |  |  |  | 7 | 86 | 440 | 1,559 | 2,989 | 4,142 | 3,224 | 843 | **0+12** |
|  | **0.8-0.9** |  |  |  |  |  | 50 | 268 | 1,085 | 3,193 | 6,200 | 5,637 | **3+548** |
|  | **0.9-0.99** |  |  |  |  |  |  | 3 | 69 | 691 | 3,582 | 14,874 | **51+6,427** |
|  | **1** |  |  |  |  |  |  |  | **2** | **49** | **750** | **12,889** | 41,677 |

“0” row: “top edge” of the table, excluded alleles (frequency most from 0.0-0.2 in LR_NC_); “0” column: “left edge” of the table, emerged and directly inherited (from WA or LR_SC_) alleles (frequency most from 0.0-0.1 in LR_NEC_); “1” row: “bottom edge” of the table, excluded loci (frequency most from 0.9-0.99 in LR_NC_); “1” column: “right edge” of the table, emerged and restored loci (frequency most from 0.9-0.99 in LR_NEC_).

Boldface signifies the important outcomes.

**Table S14. The frequency distribution of alleles from LR_SC_ to RC_SC_.**

| **Allele number** | | **Class limit of allele frequency in LR_SC_** | | | | | | | | | | | |
| --- | --- | --- | --- | --- | --- | --- | --- | --- | --- | --- | --- | --- | --- |
|  |  | **0** | **0.0-0.1** | **0.1-0.2** | **0.2-0.3** | **0.3-0.4** | **0.4-0.5** | **0.5-0.6** | **0.6-0.7** | **0.7-0.8** | **0.8-0.9** | **0.9-0.99** | **1** |
| **Class limit of allele frequency in RC_SC_** | **0** | 89,263 | **100,408** | **454** | **12** |  |  |  |  |  |  |  |  |
|  | **0.0-0.1** | **380+6,999** | 111,885 | 17,220 | 2,510 | 311 | 12 |  |  |  |  |  |  |
|  | **0.1-0.2** | **12+22** | 12,557 | 13,199 | 6,794 | 2,555 | 356 | 33 | 1 |  |  |  |  |
|  | **0.2-0.3** |  | 2,690 | 6,672 | 7,757 | 4,886 | 2,061 | 413 | 57 |  |  |  |  |
|  | **0.3-0.4** |  | 220 | 2,060 | 4,030 | 4,167 | 2,889 | 1,297 | 456 | 61 |  |  |  |
|  | **0.4-0.5** |  | 9 | 543 | 2,377 | 3,325 | 3,362 | 2,350 | 1,271 | 295 | 9 |  |  |
|  | **0.5-0.6** |  |  | 59 | 436 | 1,592 | 2,500 | 2,466 | 2,257 | 1,085 | 191 | 3 |  |
|  | **0.6-0.7** |  |  | 2 | 124 | 611 | 1,546 | 2,410 | 2,949 | 1,956 | 869 | 62 |  |
|  | **0.7-0.8** |  |  |  |  | 92 | 543 | 1,779 | 3,366 | 3,757 | 2,561 | 849 |  |
|  | **0.8-0.9** |  |  |  |  | 1 | 23 | 281 | 1,325 | 3,311 | 4,691 | 3,448 | **0+6** |
|  | **0.9-0.99** |  |  |  |  |  |  | 7 | 237 | 1,115 | 5,814 | 28,703 | **23+1,295** |
|  | **1** |  |  |  |  |  |  |  |  | **7** | **78** | **29,914** | 27,027 |

“0” row: “top edge” of the table, excluded alleles (frequency most from 0.0-0.1 in LR_SC_); “0” column: “left edge” of the table, emerged and directly inherited (from WA or LR) alleles (frequency most from 0.0-0.1 in RC_SC_); “1” row: “bottom edge” of the table, excluded loci (frequency most from 0.9-0.99 in LR_SC_); “1” column: “right edge” of the table, emerged and restored loci (frequency most from 0.9-0.99 in RC_SC_).

Boldface signifies the important outcomes.

**Table S15. The frequency distribution of alleles from LR_NC_ to RC_NC_.**

| **Allele number** | | **Class limit of allele frequency in LR_NC_** | | | | | | | | | | | |
| --- | --- | --- | --- | --- | --- | --- | --- | --- | --- | --- | --- | --- | --- |
|  |  | **0** | **0.0-0.1** | **0.1-0.2** | **0.2-0.3** | **0.3-0.4** | **0.4-0.5** | **0.5-0.6** | **0.6-0.7** | **0.7-0.8** | **0.8-0.9** | **0.9-0.99** | **1** |
| **Class limit of allele frequency in RC_NC_** | **0** | 146,850 | **53,770** | **2,386** | **134** | **1** |  |  |  |  |  |  |  |
|  | **0.0-0.1** | **319+19,944** | 65,058 | 20,344 | 5,917 | 1,314 | 257 | 12 | 1 |  |  |  |  |
|  | **0.1-0.2** | **138+1,446** | 16,251 | 13,125 | 8,180 | 4,030 | 1,488 | 332 | 26 | 1 |  |  |  |
|  | **0.2-0.3** | **6+183** | 3,645 | 6,838 | 6,941 | 4,441 | 2,967 | 1,072 | 266 | 34 | 2 |  |  |
|  | **0.3-0.4** | **0+4** | 699 | 2,666 | 4,422 | 3,162 | 3,138 | 1,553 | 581 | 201 | 17 | 3 |  |
|  | **0.4-0.5** | **0+2** | 211 | 1,167 | 2,902 | 2,888 | 3,442 | 2,574 | 1,225 | 619 | 114 | 13 |  |
|  | **0.5-0.6** |  | 126 | 328 | 1,253 | 1,912 | 3,082 | 2,850 | 1,770 | 1,384 | 420 | 53 |  |
|  | **0.6-0.7** |  | 51 | 52 | 348 | 829 | 1,668 | 2,476 | 2,059 | 2,084 | 972 | 170 |  |
|  | **0.7-0.8** |  | 4 | 13 | 67 | 342 | 1,232 | 2,333 | 2,825 | 3,120 | 2,377 | 793 | **0+46** |
|  | **0.8-0.9** |  |  |  | 18 | 28 | 343 | 1,104 | 2,145 | 3,150 | 4,267 | 3,706 | **9+263** |
|  | **0.9-0.99** |  |  |  |  | 1 | 4 | 112 | 716 | 2,244 | 6,339 | 15,726 | **22+4,539** |
|  | **1** |  |  |  |  |  |  |  | **1** | **38** | **746** | **13,890** | 43,839 |

“0” row: “top edge” of the table, excluded alleles (frequency most from 0.0-0.2 in LR_NC_); “0” column: “left edge” of the table, emerged and directly inherited (from WA or LR) alleles (frequency most from 0.0-0.1 in RC_NC_); “1” row: “bottom edge” of the table, excluded loci (frequency most from 0.9-0.99 in LR_NC_); “1” column: “right edge” of the table, emerged and restored loci (frequency most from 0.9-0.99 in RC_NC_).

Boldface signifies the important outcomes.

**Table S16. The frequency distribution of alleles from LR_NEC_ to RC_NEC_.**

| **Allele number** | | **Class limit of allele frequency in LR_NEC_** | | | | | | | | | | | |
| --- | --- | --- | --- | --- | --- | --- | --- | --- | --- | --- | --- | --- | --- |
|  |  | **0** | **0.0-0.1** | **0.1-0.2** | **0.2-0.3** | **0.3-0.4** | **0.4-0.5** | **0.5-0.6** | **0.6-0.7** | **0.7-0.8** | **0.8-0.9** | **0.9-0.99** | **1** |
| **Class limit of allele frequency in RC_NEC_** | **0** | 168,942 | **50,678** | **11,075** | **1,741** | **212** | **21** |  |  |  |  |  |  |
|  | **0.0-0.1** | **179+25,516** | 33,593 | 21,229 | 8,754 | 2,776 | 782 | 166 | 13 | 1 |  |  |  |
|  | **0.1-0.2** | **241+3,357** | 9,915 | 10,556 | 6,454 | 4,183 | 1,878 | 763 | 241 | 58 | 1 |  |  |
|  | **0.2-0.3** | **94+787** | 3,523 | 5,762 | 5,034 | 3,432 | 2,210 | 1,331 | 684 | 197 | 43 | 31 |  |
|  | **0.3-0.4** | **17+188** | 1,284 | 3,033 | 3,349 | 3,278 | 2,479 | 1,578 | 1,094 | 546 | 103 | 21 |  |
|  | **0.4-0.5** | **0+34** | 610 | 1,961 | 2,435 | 2,628 | 2,346 | 1,880 | 1,491 | 829 | 348 | 39 |  |
|  | **0.5-0.6** | **0+2** | 130 | 737 | 1,294 | 1,809 | 1,735 | 1,572 | 1,166 | 858 | 503 | 106 | **0+3** |
|  | **0.6-0.7** | **0+1** | 132 | 378 | 1,125 | 1,550 | 1,695 | 2,173 | 1,936 | 1,535 | 858 | 265 | **0+33** |
|  | **0.7-0.8** |  | 31 | 139 | 464 | 1,043 | 1,324 | 1,753 | 2,195 | 2,330 | 2,037 | 829 | **3+174** |
|  | **0.8-0.9** |  |  | 13 | 185 | 410 | 732 | 1,489 | 2,450 | 2,920 | 3,470 | 2,501 | **18+795** |
|  | **0.9-0.99** |  |  |  | 5 | 33 | 209 | 558 | 1,895 | 3,401 | 6,238 | 8,089 | **10+5,400** |
|  | **1** |  |  |  |  |  | **1** | **9** | **81** | **627** | **3,383** | **13,816** | 48,931 |

“0” row: “top edge” of the table, excluded alleles (frequency most from 0.0-0.3 in LR_NEC_); “0” column: “left edge” of the table, emerged and directly inherited (from WA or LR) alleles (frequency most from 0.0-0.1 in RC_NEC_); “1” row: “bottom edge” of the table, excluded loci (frequency most from 0.8-0.99 in LR_NEC_); “1” column: “right edge” of the table, emerged and restored loci (frequency most from 0.9-0.99 in RC_NEC_).

Boldface signifies the important outcomes.

**Table S17. Soybean seed weight/size genes identified by selective sweeps and GLAC in domestication and modern breeding (LR vs. WA and RC vs. LR).**

| **Gene number** | **Gene name** | **Selective sweep** | |  | **GLAC (LR vs. WA + RC vs. LR)** | |
| --- | --- | --- | --- | --- | --- | --- |
|  |  | **Yes** | **Location** |  | **Yes** | **SNPLDB** |
| Glyma.01G061100 | *GmCYP78A70* | √ | [1-1] |  | √ | BLK_1_8311175_8450411 |
| Glyma.02G119600 | *GmCYP78A57* |  |  |  | √ | BLK_2_11775589_11790128 |
| Glyma.02G190000 | *GmSDP1-1* | √ | [2-1] |  | √ | BLK_2_35595601_35795600 |
| Glyma.03G056700 | *GmFAD3A* |  |  |  | √ | BLK_3_7767312_7809610 |
| Glyma.05G019200 | *GmCYP78A5* | √ | (5-1) [5-1] |  | √ | BLK_5_1714275_1722100 |
| Glyma.05G244100 | *GmST05* |  |  |  | √ | BLK_5_41849526_41854422 |
|  |  |  |  |  |  | Chr05_41854447 |
|  |  |  |  |  |  | BLK_5_41854501_41863696 |
| Glyma.06G205800 | *GmFULa* |  |  |  | √ | BLK_6_19576602_19648076 |
| Glyma.06G207300 | *PG031* |  |  |  | √ | BLK_6_19940597_20013133 |
| Glyma.07G033800 | *GmGA3ox1* |  |  |  | √ | BLK_7_2643811_2644928 |
|  |  |  |  |  |  | BLK_7_2645117_2645490 |
|  |  |  |  |  |  | BLK_7_2645765_2645913 |
|  |  |  |  |  |  | BLK_7_2646143_2649816 |
| Glyma.07G081700 | *GmGA20OX* |  |  |  | √ | BLK_7_7517797_7523486 |
| Glyma.07G151300 | *GmFAD3B* |  |  |  | √ | BLK_7_18347959_18405642 |
| Glyma.08G109100 | *ST1* |  |  |  | √ | BLK_8_8375666_8382040 |
| Glyma.08G183500 | *GmSWEET10b* |  |  |  |  |  |
| Glyma.09G123600 | *GmJAZ3* |  |  |  | √ | BLK_9_29853216_30053214 |
| Glyma.10G244400 | *GmBS1* |  |  |  | √ | BLK_10_47235648_47266826 |
| Glyma.11G095200 | *hsw* |  |  |  | √ | BLK_11_7220980_7240367 |
| Glyma.11G174100 | *GmFAD3C* |  |  |  | √ | BLK_11_18965688_19019555 |
| Glyma.13G108100 | *GmPDAT* |  |  |  | √ | BLK_13_22231430_22251514 |
| Glyma.13G147800 | *GmPLATZ* |  |  |  | √ | BLK_13_26124117_26138690 |
| Glyma.15G049200 | *GmSWEET10a* | √ | [15-1] |  | √ | BLK_15_3870603_3875117 |
|  |  |  |  |  |  | Chr15_3876581 |
| Glyma.16G198300 | *GmSW16.1* |  |  |  | √ | BLK_16_35921705_35928289 |
| Glyma.17G036300 | *GmCIF1* | √ | [17-1] |  | √ | Chr17_2663704 |
|  |  |  |  |  |  | BLK_17_2663815_2664242 |
|  |  |  |  |  |  | Chr17_2664311 |
| Glyma.17G112800 | *GmKIX8-1* |  |  |  | √ | BLK_17_8906344_8911227 |
| Glyma.18G065600 | *GmFtsH25* | √ | (18-1) |  | √ | BLK_18_5986774_5993268 |
| Glyma.18G273600 | *Dt2* |  |  |  | √ | BLK_18_55644700_55646203 |
| Glyma.19G039000 | *GmCOL2b* | √ | [19-1] |  | √ | BLK_19_5447776_5469812 |
| Glyma.19G196000 | *GmSSS1* |  |  |  | √ | BLK_19_45307312_45340984 |
| Glyma.19G240800 | *GmCYP78A72* |  |  |  | √ | BLK_19_48840118_48895021 |
| Glyma.20G019300 | *GmNAP1* |  |  |  | √ | BLK_20_2000817_2023334 |
| Glyma.20G085100 | *POWR1-TE* |  |  |  | √ | BLK_20_31773021_31798296 |
| Glyma.20G196600 | *GmOLEO1* |  |  |  | √ | BLK_20_43439636_43458136 |

GLAC (LR vs. WA + RC vs. LR): allele exclusion or emergence happened in LR vs. WA and RC vs. LR identified by GLAC.

The 31 soybean seed weight/size genes reviewed in Hu *et al*. (2023). Regulation of seed traits in soybean. *aBIOTECH*, *4*(4), 372-385.

See Table S8 for the location of domestication selective sweeps, like (5-1), indicating the identified first gene on Chromosome 5.

And see Table S9 for the location of modern breeding selective sweeps, like [1-1], indicating the identified first gene on Chromosome 1.
